# Diversity, antibacterial and phytotoxic activities of culturable endophytic fungi from *Pinellia pedatisecta* and *Pinellia ternata*

**DOI:** 10.1186/s12866-022-02741-5

**Published:** 2023-01-28

**Authors:** Kun Kong, Zhongdi Huang, Shuping Shi, Weidong Pan, Yinglao Zhang

**Affiliations:** 1 School of Life Sciences, Anhui Agricultural University, Hefei, 230036 China; 2 State Key Laboratory of Functions and Applications of Medicinal Plants, Guizhou Medical University, Guiyang, 550014 China

**Keywords:** *Pinellia ternata*, *Pinellia pedatisecta*, Secondary metabolites, Antibacterial activity, Phytotoxic activity

## Abstract

**Background:**

Endophytic fungi of medicinal plants, as special microorganisms, are important sources of antibacterial compounds. However, the diversity and antibacterial activity of endophytic fungi from *Pinellia* Tenore have not been systematically studied.

**Results:**

A total of 77 fungi were isolated from roots, stems, leaves, and tubers of *Pinellia ternata* and *P. pedatisecta*. All fungi were belonged to five classes and twenty-five different genera. Biological activities tests indicated that 21 extracts of endophytic fungi exhibited antibacterial activities against at least one of the tested bacteria, and 22 fermentation broth of endophytic fungi showed strong phytotoxic activity against *Echinochloa crusgalli* with the inhibition rate of 100%. Furthermore, four compounds, including alternariol monomethyl ether (**1**), alternariol (**2**), dehydroaltenusin (**3**) and altertoxin II (**4**), and three compounds, including terreic acid (**5**), terremutin (**6**), citrinin (**7**), were isolated from *Alternaria angustiovoidea* PT09 of *P. ternata* and *Aspergillus floccosus* PP39 of *P. pedatisecta*, respectively. Compound **5** exhibited strong antibacterial activities against *Escherichia coli*, *Micrococcus tetragenus*, *Staphylococcus aureus*, and *Pseudomonas syringae* pv. *actinidiae* with the inhibition zone diameter (IZD) of 36.0, 31.0, 33.7, 40.2 mm and minimum inhibitory concentration (MIC) values of 1.56, 3.13, 1.56, 1.56 μg/mL respectively, which were better than or equal to those of positive gentamicin sulfate. The metabolite **7** also exhibited strong antibacterial activity against *P. syringae* pv. *actinidiae* with the IZD of 26.0 mm and MIC value of 6.25 μg/mL. In addition, the compound **7** had potent phytotoxic activity against *E. crusgalli* with the inhibition rate of 73.4% at the concentration of 100 μg/mL.

**Conclusions:**

Hence, this study showed that endophytic fungi of *P. ternata* and *P. pedatisecta* held promise for the development of new antibiotic and herbicide resources.

**Supplementary Information:**

The online version contains supplementary material available at 10.1186/s12866-022-02741-5.

## Background

There are five medicinal species belonging to *Pinellia* Tenore in China, including *Pinellia cordata*, *P. integrifolia*, *P. pedatisecta*, *P. peltata* and *P. ternata* [1]. Their tubers possess important medicinal values, such as antitussive and expectorant [2], antiemetic [3], antitumor [4], anti-fertility and terminate early pregnancy effects [5]. *Pinellia* Tenore also contains diverse phytochemicals, for instance, protein, lectins, alkaloids, sterols, flavonoids, and tannins [2–5]. Among them, alkaloids and lectins have been identified as the main active ingredients of *Pinellia* Tenore [6, 7]. *P. pedatisecta* and *P. ternata* are the main medicinal plants of the *Pinellia* Tenore and have similar bioactivities [6].

Endophytic fungi are widespread in the tissues of almost all plants [8], such as roots, stems and leaves [9–11]. Furthermore, endophytic fungi protect plants from pathogenic microorganisms and weeds by producing bioactive secondary metabolites [12, 13]. Secondary metabolites produced by endophytic fungi also have antibacterial, antifungal, insecticidal, herbicidal, cytotoxic, antioxidant and anticancer effects [14, 15]. For example, solanioic acid produced by *Rhizoctonia solani* isolated from *Cyperus rotundus* showed significant activity against Gram-positive bacteria (*Bacillus subtilis*, *Staphylococcus aureus*, and MRSA) [16]. Those bioactive secondary metabolites have significant potential applications in the pharmaceutical industry [14, 17].

Secondary metabolites of endophytic fungi of *P. ternata* have been shown to have outstanding antibacterial activity [18–20]. For instance, aspergillone A from endophytic *Aspergillus cristatus* of *P. ternata* exhibited remarkable antibacterial effects [20]. However, as far as we know, the diversity and antibacterial activity of its endophytic fungi have not been systematically studied. Besides, the lack of research focused on the endophytic fungi of *P. pedatisecta*. The main objective of this study is to identify the diversity of endophytic fungi from *P. ternata* and *P. pedatisecta* and to obtain endophytic fungal resources with antibacterial and herbicidal activity. This study further provides a basis for obtaining biotrophic fungi or mining bioactive natural products.

## Results

### Isolation and identification of endophytic fungi

**Fig. 1:**
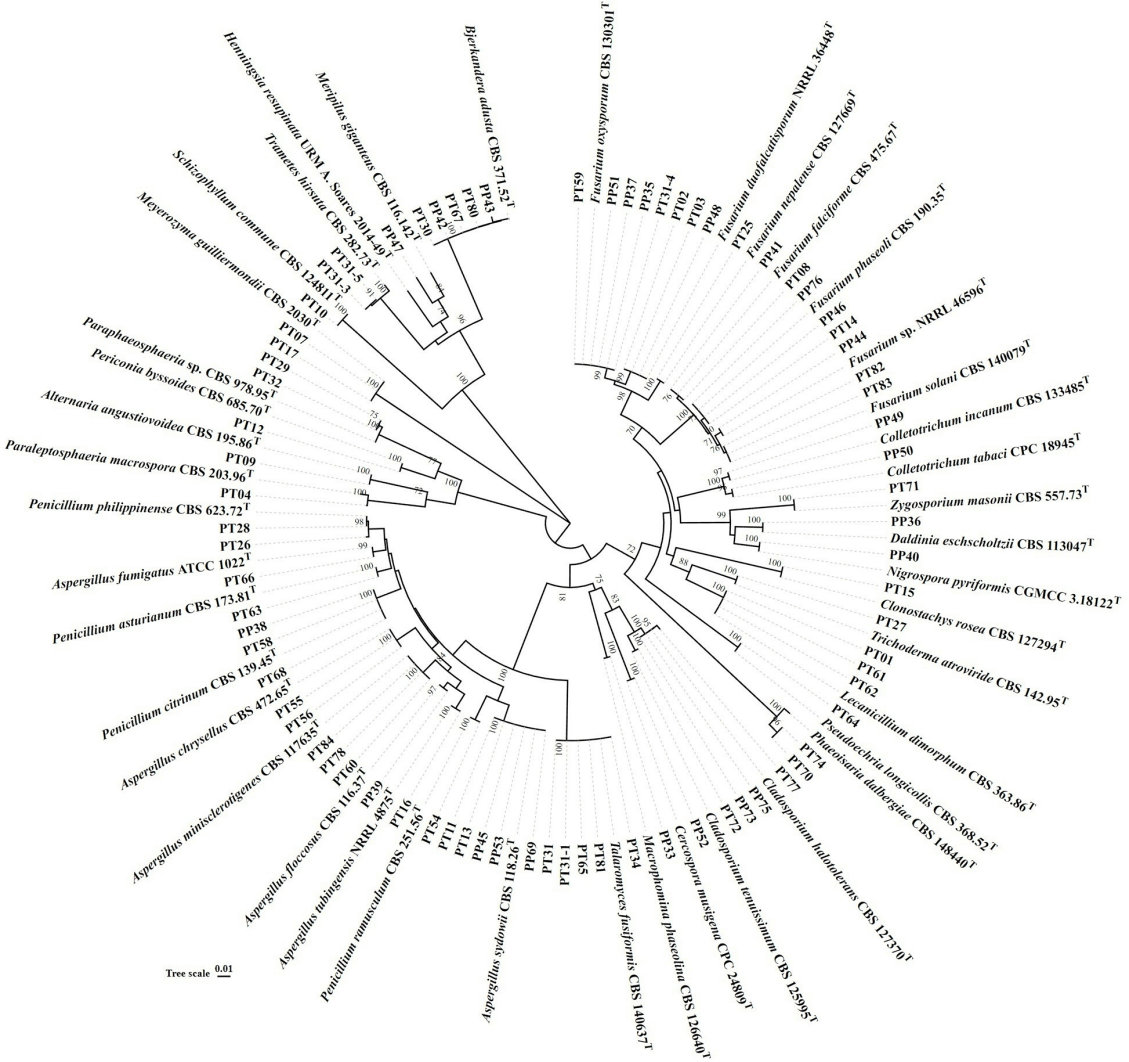
Neighbor-joining phylogenetic tree of 77 fungi isolates from *P. ternata* and *P. pedatisecta*. The phylogenetic tree based on ITS gene sequences. The values at each node represent the bootstrap values from 1000 replicates

A total of 77 strains (Table 1) of endophytic fungi were isolated from *P. ternata* and *P. pedatisecta* for the first time. There were 53 and 24 strains of fungi that were isolated from all tissues of *P. ternata* and *P. pedatisecta*, respectively. The ITS1-ITS4 region of 77 strains was sequenced and compared with available GenBank reference sequences. The obtained 5.8S rDNA sequences were uploaded to NCBI under the accession numbers ON677855-ON677931. The results of sequence analysis showed that 77 fungi were attached to the phyla Ascomycota and Basidiomycota (Fig. 1). The 68 strains were grouped into four classes [Dothideomycetes (14.3%), Eurotiomycetes (32.5%), Saccharomycetes (2.6%) and Sordariomycetes (39.0%)] within the phylum Ascomycota. Nine other strains (11.7%) were distributed in the Agaricomycetes within the phylum Basidiomycota. The fungi of Sordariomycetes were the dominant species of cultivable fungi in phylogenetic diversity from *P. ternata* and *P. pedatisecta*. The largest number (Sordariomycetes, 30) of isolates was distributed in 5 orders, including the Glomerellales, Hypocreales, Pleurotheciales, Sordariales and Xylariales.

**Table 1:** Phylogenetic analysis of cultivable fungi associated with *P. ternata* and *P. pedatisecta*

Isolate code	Source	Closest match	Accession no	Coverage/Max ident	GenBank no
PT09^a^	root	*Alternaria angustiovoidea*	MH861939.1	100/100	ON677861
PT55	leaf	*Aspergillus chrysellus*	OL711749.1	100/100	ON677904
PT56	leaf	*Aspergillus chrysellus*	OL711749.1	100/99.8	ON677905
PP39^b^	root	*Aspergillus floccosus*	KP987086.1	100/100	ON677888
PT66	tuber	*Aspergillus fumigatus*	NR121481.1	100/100	ON677914
PT60	tuber	*Aspergillus minisclerotigenes*	OL711675.1	100/99.8	ON677908
PT78	tuber	*Aspergillus minisclerotigenes*	OL711675.1	99/99.8	ON677926
PT84	tuber	*Aspergillus minisclerotigenes*	OL711675.1	100/100	ON677931
PP45	stem	*Aspergillus sydowii*	MH854859.1	100/100	ON677894
PP53	tuber	*Aspergillus sydowii*	MH854859.1	100/100	ON677902
PP69	leaf	*Aspergillus sydowii*	MH854859.1	99/99.5	ON677917
PT11	tuber	*Aspergillus sydowii*	MH854859.1	100/98.5	ON677863
PT13	tuber	*Aspergillus sydowii*	MH854859.1	100/98.5	ON677865
PT16	leaf	*Aspergillus tubingensis*	NR131293.1	100/99.8	ON677868
PP42	tuber	*Bjerkandera adusta*	MH857085.1	100/99.5	ON677891
PP43	leaf	*Bjerkandera adusta*	MH857085.1	100/99.5	ON677892
PT30	tuber	*Bjerkandera adusta*	MH857085.1	100/99.5	ON677875
PT67	tuber	*Bjerkandera adusta*	MH857085.1	99/99.6	ON677915
PT80	leaf	*Bjerkandera adusta*	MH857085.1	100/99.5	ON677927
PP52	leaf	*Cercospora musigena*	NR147294.1	100/100	ON677901
PP73	stem	*Cladosporium halotolerans*	MH864551.1	100/100	ON677921
PP75	tuber	*Cladosporium halotolerans*	MH864551.1	100/100	ON677923
PT77	tuber	*Cladosporium halotolerans*	MH864551.1	100/99.6	ON677925
PT72	tuber	*Cladosporium tenuissimum*	MH864840.1	100/100	ON677920
PT15	tuber	*Clonostachys rosea*	MH864507.1	100/99.8	ON677867
PP49	leaf	*Colletotrichum incanum*	NR160812.1	98/99.6	ON677898
PP50	leaf	*Colletotrichum tabaci*	NR144804.1	98/100	ON677899
PP36	stem	*Daldinia eschscholtzii*	KY610387.1	99/99.3	ON677885
PP48	leaf	*Fusarium duofalcatisporum*	GQ505741.1	100/99.4	ON677897
PT02	tuber	*Fusarium duofalcatisporum*	GQ505741.1	100/99.6	ON677856
PT03	tuber	*Fusarium duofalcatisporum*	GQ505741.1	100/99.6	ON677857
PP41	root	*Fusarium falciforme*	NR164424.1	96/99.8	ON677890
PT08	root	*Fusarium falciforme*	NR164424.1	100/100	ON677860
PT25	tuber	*Fusarium nepalense*	MH864615.1	99/100	ON677870
PP35	stem	*Fusarium oxysporum*	MH865885.1	100/99.8	ON677884
PP37	stem	*Fusarium oxysporum*	MH865885.1	100/99.8	ON677886
PP51	leaf	*Fusarium oxysporum*	MH865885.1	99/98.8	ON677900
PT31-4	stem	*Fusarium oxysporum*	MH865885.1	99/98.8	ON677879
PT59	leaf	*Fusarium oxysporum*	MH865885.1	99/98.8	ON677907
PP46	stem	*Fusarium phaseoli*	MH855640.1	100/98.9	ON677895
PP76	tuber	*Fusarium phaseoli*	MH855640.1	100/98.9	ON677924
PT14	tuber	*Fusarium phaseoli*	MH855640.1	100/99.1	ON677866
PT82	tuber	*Fusarium solani*	NR163531.1	99/100	ON677929
PT83	tuber	*Fusarium solani*	NR163531.1	100/100	ON677930
PP44	tuber	*Fusarium* sp.	GU170647.1	99/100	ON677893
PT01	tuber	*Lecanicillium dimorphum*	MH861964.1	100/99.7	ON677855
PT61	tuber	*Lecanicillium dimorphum*	MH861964.1	99/99.7	ON677909
PT62	tuber	*Lecanicillium dimorphum*	MH861964.1	99/99.7	ON677910
PP33	root	*Macrophomina phaseolina*	MH864182.1	100/100	ON677882
PP47	leaf	*Meripilus giganteus*	GQ355959.1	99/86.6	ON677896
PT07	stem	*Meyerozyma guilliermondii*	MH545918.1	100/100	ON677859
PT17	tuber	*Meyerozyma guilliermondii*	MH545918.1	100/100	ON677869
PP40	tuber	*Nigrospora pyriformis*	NR153469.1	97/98.2	ON677889
PT04	root	*Paraleptosphaeria macrospora*	MH862571.1	98/97.2	ON677858
PT29	stem	*Paraphaeosphaeria* sp.	JX496120.1	100/98.1	ON677874
PT32	stem	*Paraphaeosphaeria* sp.	JX496120.1	100/98.1	ON677881
PT63	tuber	*Penicillium asturianum*	MH861321.1	100/100	ON677911
PP38	root	*Penicillium citrinum*	MH856132.1	97/100	ON677887
PT58	leaf	*Penicillium citrinum*	MH856132.1	99/100	ON677906
PT68	leaf	*Penicillium citrinum*	MH856132.1	100/100	ON677916
PT26	root	*Penicillium philippinense*	MH860600.1	99/98.4	ON677871
PT28	root	*Penicillium philippinense*	MH860600.1	99/98.4	ON677873
PT54	leaf	*Penicillium ramusculum*	MH857613.1	100/100	ON677903
PT12	stem	*Periconia byssoides*	MH859902.1	100/99.3	ON677864
PT70	stem	*Phaeoisaria dalbergiae*	NR175205.1	99/98.0	ON677918
PT74	stem	*Phaeoisaria dalbergiae*	NR175205.1	100/97.8	ON677922
PT64	tuber	*Pseudoechria longicollis*	NR145145.1	99/100	ON677912
PT10	root	*Schizophyllum commune*	MH863418.1	100/100	ON677862
PT31	stem	*Talaromyces fusiformis*	NR169911.1	100/100	ON677876
PT31-1	stem	*Talaromyces fusiformis*	NR169911.1	100/100	ON677877
PT34	tuber	*Talaromyces fusiformis*	NR169911.1	100/100	ON677883
PT65	tuber	*Talaromyces fusiformis*	NR169911.1	100/100	ON677913
PT81	tuber	*Talaromyces fusiformis*	NR169911.1	100/100	ON677928
PT31-3	stem	*Trametes hirsuta*	MH860685.1	100/98.6	ON677878
PT31-5	stem	*Trametes hirsuta*	MH860685.1	100/98.7	ON677880
PT27	tuber	*Trichoderma atroviride*	AF456917.1	100/99.8	ON677872
PT71	tuber	*Zygosporium masonii*	MH860771.1	100/99.6	ON677919

^a^*PT Pinellia ternata*

^b^*PP Pinellia pedatisecta*

There were 10, 17, 16 and 34 strains, which belonged to 7, 9, 7 and 13 genera, from roots, stems, leaves, and tubers, respectively. Among them, *Alternaria angustiovoidea*, *Macrophomina phaseolina* and *Schizophyllum commune* were isolated from their roots only. Similarly, *Clonostachys rosea*, *Lecanicillium dimorphum*, *Nigrospora pyreformis*, *Pseudoechria longicollis*, *Trichoderma atroviride* and *Zygosporium masonii* were isolated from tubers only. Therefore, the results of phylogenetic diversity showed that the endophytic fungi were different in different plant tissues.

### Diversity analyses of endophytic fungi from *P. ternata* and *P. pedatisecta*

The diversity analyses of endophytic fungi genera isolated from *P. ternata* and *P. pedatisecta* were summarized and shown in Table 2. The Species richness (*S*) and Margalef index (*D'*) values showed positive correlation with endophytic fungal genera [21]. The higher the Shannon–Wiener index (*H'*) and the Simpson diversity index (*D*_*s*_), the more diverse the microbial community [22]. Therefore, the endophytic fungi of tubers from *P. ternata* showed high species richness and diversity, with the values of *S* (12), *D'* (3.3011), *H'* (2.2299), *D*_*s*_ (0.8673), and *PIE* (0.8995). However, the endophytic fungi of the leaf from *P. pedatisecta* had high species richness and diversity with the values of *S* (6), *D'* (2.4045), *H'* (1.7329), *D*_*s*_ (0.8125), and *PIE* (0.9286). The Pielou indexes (*J*) could reflect the level of uniformity in the distribution of the number of individuals of the species in the community [23]. In this study, the endophytic fungi of stem from *P. ternata* and tuber from *P. pedatisecta* showed high Pielou indexes (*J*) with values of 0.9732 and 0.9697, respectively, which indicated a convergence in the number of individuals of each species.

**Table 2:** Diversity analyses of endophytic fungi from *P. ternata* and *P. pedatisecta*

**Diversity Index**	***Pinellia ternata***				***Pinellia pedatisecta***			
	**Root**	**Stem**	**Leaf**	**Tuber**	**Root**	**Stem**	**Leaf**	**Tuber**
Species richness (*S*)	5	7	4	12	4	4	6	5
Margalef index (*D'*)	2.7906	2.5022	1.4427	3.3011	2.1640	1.6743	2.4045	2.2324
Shannon–Wiener index (*H'*)	1.5607	1.8938	1.2555	2.2299	1.3863	1.2425	1.7329	1.5607
Simpson diversity index (*D*_*s*_)	0.7778	0.8430	0.6875	0.8673	0.7500	0.6667	0.8125	0.7778
PIE index (*PIE*)	0.9333	0.9273	0.7857	0.8995	1.0000	0.8000	0.9286	0.9333
Dominant index (*λ*)	0.2222	0.1570	0.3125	0.1327	0.2500	0.3333	0.1875	0.2222
Pielou index (*J*)	0.9697	0.9732	0.9056	0.8974	1.0000	0.8962	0.9671	0.9697

### Antibacterial activities of the crude extracts of fungi

The filter paper disk method was used to evaluate the antibacterial activity of 77 fungal extracts from *P. ternata* and *P. pedatisecta*. The results showed that 21 extracts (27.3%) showed antibacterial activities against at least one pathogenic bacterium (Supplementary Table S1). Five of them (PT02, PP35, PP37, PP39, and PT58) had inhibitory activities against all four pathogens (*Escherichia coli*, *Micrococcus tetragenus*, *Staphylococcus aureus*, and *Pseudomonas syringae* pv*. actinidiae*). Among them, PP39 exhibited strong antibacterial activity against *S. aureus* with an IZD of 20.0 mm, which was equivalent to that of positive gentamicin sulfate with an IZD of 21.7 mm. PP39 also showed potent inhibition activities against *M. tetragenus*, *E. coli* and *P. syringae* pv. *actinidiae* with the IZD of 14.2, 15.2, and 14.0 mm, which were weaker than those of positive gentamicin sulfate with the IZD of 25.7, 26.7, and 24.3 mm, respectively. Besides, the strain PT83 showed strong inhibition activity against *P. syringae* pv. *actinidiae* with an IZD of 20.0 mm, which was comparable to that of positive gentamicin sulfate. In addition, the crude extracts of strains PT56 and PT82 also exhibited potent antibacterial activities against *P. syringae* pv. *actinidiae* with the IZD of 15.2 mm and 15.7 mm, respectively.

### Phytotoxic assay

As the results shown in Table 3, 52 fermentation broth of endophytic fungi exhibited significant phytotoxic activity against the radicle of *Echinochloa crusgalli* with the inhibition rate of more than 50%. Among them, 22 strains showed strong phytotoxic activity against *E. crusgalli* with the inhibition rate of 100%. Interestingly, these fungal strains were mainly assigned to three genera (*Aspergillus*, *Fusarium*, and *Talaromyces*) and most of them (20 strains) were isolated from *P. ternata*. Besides, 16 strains showed outstanding phytotoxic activity against *E. crusgalli* with the inhibition rate of 80%-99%. Moreover, nine strains showed potent phytotoxic activity against *E. crusgalli* with the inhibition rate of 60%-79%. In addition, 25 strains showed relatively weak phytotoxic activity against *E. crusgalli* with the inhibition rate of 10%-60%.

**Table 3:** Inhibitory activity of the fermentation broth of 77 endophytic fungi of *P. ternata* and *P. pedatisecta* on the radicle growth of *E. crusgalli*

Strains	Inhibition rate /%	Strains	Inhibition rate /%	Strains	Inhibition rate /%
PT01	100.0 ± 0.0^*^	PT32	81.9 ± 3.1^*, #^	PT84	100.0 ± 0.0^*^
PT02	NI^a, #^	PT34	59.0 ± 3.6^*, #^	PP33	NI^#^
PT03	30.7 ± 5.9^*, #^	PT54	87.6 ± 1.8^*, #^	PP35	44.1 ± 6.8^*, #^
PT04	67.9 ± 5.4^*, #^	PT55	100.0 ± 0.0^*^	PP36	13.4 ± 2.9^*, #^
PT07	100.0 ± 0.0^*^	PT56	100.0 ± 0.0^*^	PP37	100.0 ± 0.0^*^
PT08	100.0 ± 0.0^*^	PT58	96.0 ± 4.3^*^	PP38	72.0 ± 5.8^*, #^
PT09	91.1 ± 4.3^*, #^	PT59	100.0 ± 0.0^*^	PP39	100.0 ± 0.0^*^
PT10	43.4 ± 5.4^*, #^	PT60	95.9 ± 6.3^*^	PP40	93.8 ± 6.0^*, #^
PT11	100.0 ± 0.0^*^	PT61	98.2 ± 4.0^*^	PP41	34.5 ± 4.6^*, #^
PT12	45.3 ± 4.9^*, #^	PT62	83.1 ± 6.3^*, #^	PP42	95.5 ± 4.8^*^
PT13	62.7 ± 5.3^*, #^	PT63	98.0 ± 3.1^*^	PP43	65.7 ± 5.6^*, #^
PT14	100.0 ± 0.0^*^	PT64	69.6 ± 6.2^*, #^	PP44	35.0 ± 5.4^*, #^
PT15	92.3 ± 5.9^*, #^	PT65	100.0 ± 0.0^*^	PP45	NI^#^
PT16	100.0 ± 0.0^*^	PT66	87.5 ± 3.9^*, #^	PP46	23.0 ± 5.2^*, #^
PT17	100.0 ± 0.0^*^	PT67	48.8 ± 7.0^*, #^	PP47	77.5 ± 4.1^*, #^
PT25	100.0 ± 0.0^*^	PT68	85.9 ± 5.9^*, #^	PP48	23.8 ± 3.6^*, #^
PT26	54.5 ± 6.6^*, #^	PT70	65.7 ± 4.1^*, #^	PP49	NI^#^
PT27	47.5 ± 5.8^*, #^	PT71	100.0 ± 0.0^*^	PP50	15.2 ± 4.4^*, #^
PT28	22.9 ± 5.6^*, #^	PT72	96.8 ± 4.7^*^	PP51	85.2 ± 3.7^*, #^
PT29	100.0 ± 0.0^*^	PT74	65.4 ± 2.9^*, #^	PP52	13.4 ± 3.3^*, #^
PT30	33.1 ± 5.0^*, #^	PT77	100.0 ± 0.0^*^	PP53	NI^#^
PT31	31.8 ± 5.8^*, #^	PT78	100.0 ± 0.0^*^	PP69	78.5 ± 5.0^*, #^
PT31-1	100.0 ± 0.0^*^	PT80	57.1 ± 6.2^*, #^	PP73	11.0 ± 4.5^*, #^
PT31-3	52.0 ± 4.9^*, #^	PT81	100.0 ± 0.0^*^	PP75	54.4 ± 6.5^*, #^
PT31-4	31.9 ± 5.7^*, #^	PT82	100.0 ± 0.0^*^	PP76	16.5 ± 6.1^*, #^
PT31-5	49.1 ± 3.4^*, #^	PT83	81.6 ± 4.9^*, #^		

Results were presented as the mean ± standard deviation for triplicate experiments; a: NI = not inhibited; ^*^p < 0.05, significantly different from the control; ^#^*p* < 0.05, significantly different from the strain PT01

### Identification of the secondary metabolites isolated from PT09 and PP39

The fermentation broth PP39 from *P. pedatisecta* had the best antibacterial and phytotoxic activities among all tested endophytic fungi. PT09 from *P. ternata* also had strong phytotoxic activity against *E. crusgalli* with the inhibition rate of 91.1% and moderate antibacterial activity against *S. aureus* and *P. syringae* pv. *actinidiae*. Therefore, both PT09 and PP39 were selected as further research objects of active secondary metabolites.

**Fig. 2:**
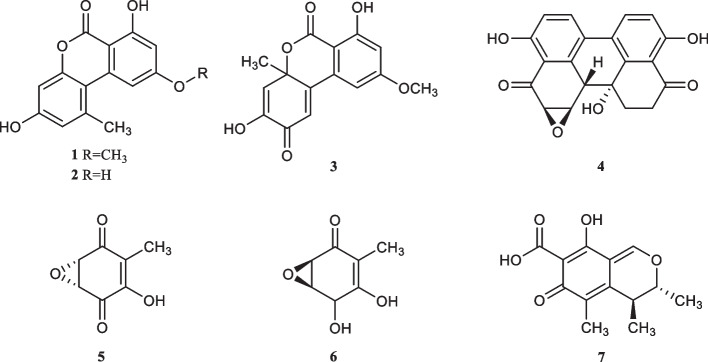
The structure of compounds **1**–**7**

Four monomer compounds (Fig. 2) were isolated from the liquid fermentation product of strain PT09. They were further identified as alternariol monomethyl ether (**1**), alternariol (**2**), dehydroaltenusin (**3**) and altertoxin II (**4**) by spectroscopic analyses, including HR–ESI–MS, NMR, and compared with data described in previous literature.

Alternariol monomethyl ether (**1**) (Figures S1-S2): colorless crystal; HR-ESI–MS: m/z: 271.0599 [M—H]^−^, calculated for C_15_H_11_O_5_ 271.0621; ^1^H NMR (600 MHz, DMSO*-d*_*6*_) *δ*: 11.81 (s, OH-7), 10.35 (br s, OH-3), 7.20 (s, H-10), 6.72 (s, H-4), 6.63 (s, H-2), 6.60 (s, H-8), 3.90 (s, 3H, OCH_3_-9), 2.72 (s, 3H, H-11). Due to the chemical shifts and relative molecular masses in agreement with reported in the literature [24], the structure of the compound was determined to be alternariol monomethyl ether.

Alternariol (**2**) (Figures S3-S4) [25]: white crystal; HR-ESI–MS: m/z: 257.0453 [M—H]^−^, calculated for C_14_H_9_O_5_ 257.0464; ^1^H NMR (600 MHz, Acetone-*d*_*6*_) *δ*: 11.93 (s, OH-3), 9.69 (br s, OH-5), 9.21 (br s, OH-4’), 7.34 (d, *J* = 2.4 Hz, H-6), 6.78 (d, *J* = 2.7 Hz, H-5’), 6.69 (d, *J* = 2.7 Hz, H-3’), 6.44 (d, *J* = 2.4 Hz, H-4), 2.76 (s, 3H, CH_3_-6’).

Dehydroaltenusin (**3**) (Figures S5-S7) [26]: yellow green solid; HR-ESI–MS: m/z: 287.0558 [M—H]^−^, calculated for C_15_H_11_O_6_ 287.0570; ^1^H NMR (600 MHz, CDCl_3_) *δ*: 11.29 (s, OH-7), 6.73 (d, *J* = 2.3 Hz, H-10), 6.69 (s, H-1), 6.63 (d, *J* = 2.3 Hz, H-8), 6.41 (s, H-3), 6.28 (s, H-4), 3.91 (s, 3H, OCH_3_-9), 1.73 (s, 3H, CH_3_-4a); ^13^C NMR (150 MHz, CDCl_3_) *δ*: 180.9 (C-2), 167.5 (C-6), 166.5 (C-9), 164.9 (C-7), 153.3 (C-10b), 146.3 (C-3), 135.2 (C-10a), 121.0 (C-1), 116.3 (C-4), 104.5 (C-10), 103.9 (C-8), 100.0 (C-6a), 79.3 (C-4a), 56.1 (OCH_3_-9), 29.6 (CH_3_-4a).

Altertoxin II (**4**) (Figures S8-S10) [27]: white crystal; HR-ESI–MS: m/z: 351.0849 [M + H]^+^, calculated for C_20_H_15_O_6_ 351.0854; ^1^H NMR (600 MHz, CDCl_3_) *δ*: 12.71 (s, OH-18), 12.12 (s, OH-13), 7.91 (d, *J* = 8.8 Hz, H-20), 7.86 (d, *J* = 8.8 Hz, H-11), 7.11 (d, *J* = 8.8 Hz, H-19), 7.06 (d, *J* = 8.7 Hz, H-12), 4.23 (d, *J* = 3.7 Hz, H-10), 3.71 (d, *J* = 3.5 Hz, H-9), 3.54 (s, H-6), 3.30 – 3.21 (m, H-15), 2.89 (m, H-14),2.83 (m, H-15), 2.41 (td, *J* = 13.6, 4.0 Hz, H-14); ^13^C NMR (150 MHz, CDCl_3_) *δ*: 204.3 (C-16), 196.8 (C-8), 163.5 (C-18), 162.9 (C-13), 139.0 (C-5), 133.7 (C-2), 133.0 (C-20), 132.7 (C-11), 124.1 (C-3), 122.6 (C-4), 120.0 (C-19), 118.2 (C-12), 114.8 (C-17), 113.7 (C-7), 68.5 (C-1), 55.9 (C-10), 52.9 (C-9), 45.3 (C-6), 33.4 (C-14), 32.3 (C-15).

Three monomeric compounds (Fig. 2) were isolated from the liquid fermentation product of strain PP39. The compounds were further analyzed and identified as terreic acid (**5**), terremutin (**6**), citrinin (**7**) by spectroscopic analysis, including HR–ESI–MS, NMR, and compared with data described in previous literature.

Terreic acid (**5**) (Figures S11-S13) [28]: white crystal; HR-ESI–MS: m/z: 153.0193 [M—H]^−^, calculated for C_7_H_5_O_4_ 153.0202; ^1^H NMR (600 MHz, CDCl_3_) *δ*: 1.93 (s, 3H, 3-Me), 3.87 (d, *J* = 3.7 Hz, H-6), 3.90 (d, *J* = 3.5 Hz, H-5), 6.83 (s, 2-OH); ^13^C-NMR (150 MHz, CDCl_3_) *δ*: 190.78 (C-4), 187.66 (C-1), 152.03 (C-2), 120.57 (C-3), 53.97 (C-5), 51.74 (C-6), 8.88 (3-Me).

Terremutin (**6**) (Figures S14-S16) [29]: white crystal; HR-ESI–MS: m/z: 157.0495 [M + H]^+^, calculated for C_7_H_9_O_4_ 157.0487; ^1^H NMR (600 MHz, Acetone*-d*_*6*_) *δ*: 1.65 (s, 3H, H-7), 3.35 (s, H-6), 3.64 (s, H-5), 4.60 (s, H-4); ^13^C-NMR (150 MHz, Acetone*-d*_*6*_) *δ*: 109.0 (C-2), 66.3 (C-4), 55.3 (C-6), 52.3 (C-5), 7.5 (C-7).

Citrinin (**7**) (Figures S17-S19) [30]: yellow crystal; HR-ESI–MS: m/z: 251.0913 [M + H]^+^, calculated for C_13_H_15_O_5_ 251.0905; ^1^H NMR (600 MHz, CDCl_3_) *δ*: 1.23 (d, *J* = 7.3 Hz, 3H, H-10), 1.34 (d, *J* = 6.8 Hz, 3H, H-9), 2.02 (s, 3H, H-11), 2.98 (q, *J* = 7.3 Hz, H-4), 4.77 (q, *J* = 6.5 Hz, H-3), 8.23 (s, H-1); ^13^C-NMR (150 MHz, CDCl_3_) *δ*: 184.0 (C-6), 177.4 (C-8), 174.7 (C-12), 162.7 (C-1), 139.1 (C-4a), 123.3 (C-5), 107.7 (C-8a), 100.6 (C-7), 81.8 (C-3), 34.8 (C-4), 18.6 (C-9), 18.4 (C-10), 9.6 (C-11).

### Antibacterial activities of secondary metabolites isolated from PT09 and PP39

The antibacterial activities of seven compounds isolated from strains PT09 and PP39 were tested as shown in Table 4. The results showed that compound **5** exhibited strong antibacterial activities against *E. coli*, *M. tetragenus*, *S. aureus*, and *P. syringae* pv. *actinidiae* with the IZD of 36.0, 31.0, 33.7, 40.2 mm and MIC values of 1.56, 3.13, 1.56, 1.56 μg/mL, which were better than or equal to those of positive gentamicin sulfate with the IZD of 26.7, 25.7, 21.7, 26.0 mm and MIC values of 3.13, 3.13, 1.56, 6.25 μg/mL, respectively. The metabolite **7** also exhibited strong antibacterial activity against *P. syringae* pv. *actinidiae* with the IZD of 26.0 mm and MIC value of 6.25 μg/mL, and moderate activity against *S. aureus* with the IZD of 10.0 mm and MIC value of 100 μg/mL. Moreover, compound **4** showed potent or moderate antibacterial activity against *P. syringae* pv. *actinidiae* and *S. aureus* with the IZD of 16.2, 13.3 mm, and MIC values of 25, 100 μg/mL, respectively. In addition, compounds **2** and **3** showed moderate antibacterial activities against *P. syringae* pv. *actinidiae* and *S. aureus* with the IZD of 15.3 mm and 14.2 mm, respectively. However, they were found to have MIC values of more than 100 μg/mL on both pathogenic bacteria. Besides, compounds **1** and **6** were found to have no effect on four tested pathogenic bacteria.

**Table 4:** IZD (mm) and MIC(μg/mL) of compounds **1**–**7** against the tested bacteria

Compounds	*S. aureus*		*M. tetragenus*		*E. coli*		*P. syringae* pv. *actinidiae*	
	MIC	IZD	MIC	IZD	MIC	IZD	MIC	IZD
**1**	> 100	NI^b^	> 100	NI	> 100	NI	> 100	NI
**2**	> 100	NI	> 100	NI	> 100	NI	> 100	15.3 ± 0.6^*, #^
**3**	> 100	14.2 ± 0.5^*, #^	> 100	NI	> 100	NI	> 100	NI
**4**	100	13.3 ± 0.2^*, #^	> 100	NI	> 100	NI	25	16.2 ± 0.6^*, #^
**5**	1.56	33.7 ± 0.5^*^	3.13	31.0 ± 0.8^*^	1.56	36.0 ± 0.8	1.56	40.2 ± 0.7^*^
**6**	> 100	NI	> 100	NI	> 100	NI	> 100	NI
**7**	100	10.0 ± 0.7^*, #^	> 100	NI	> 100	NI	6.25	26.0 ± 0.5^#^
Gentamicin sulfate^a^	1.56	21.7 ± 0.6^#^	3.13	25.7 ± 0.9^#^	3.13	26.7 ± 0.5^#^	6.25	26.0 ± 1.0^#^

Results were presented as the mean ± standard deviation for triplicate experiments; a: Gentamicin sulfate = positive control; b: NI = not inhibited; the concentration for the test is 30 μg/filter paper; ^*^p < 0.05, significantly different from the control; ^#^*p* < 0.05, significantly different from the compound **5**

### Phytotoxic assay of secondary metabolites isolated from PT09 and PP39

Metabolites **1**–**7** were assayed for their ability to inhibit radicle growth of *E. crusgalli* and *Abutilon theophrasti* using a Petri dish bioassay (Table 5). The results showed that metabolite **7** had potent phytotoxic activity against *E. crusgalli* and *A. theophrasti* with the inhibition rate of 73.4% and 41.7%, respectively, which was weaker than those of the positive 2,4-D with an inhibition rate of 100% at a concentration of 100 μg/mL. Compound **5** had moderate phytotoxic activity against *E. crusgalli* and *A. theophrasti* with the inhibition rates of 38.4% and 38.0%, respectively. However, metabolites **1**–**4** and **6** showed weak inhibitory activity in this bioassay with the inhibition rate of less than 31%.

**Table 5:** Inhibition rate (%) of compounds **1**–**7** on the radicle growth of *E. crusgalli* and *A. theophrasti*

Compounds	*E. crusgalli*	*A. theophrasti*
**1**	NI^b, #^	22.1 ± 5.3^*, #^
**2**	18.8 ± 4.3^*, #^	30.1 ± 5.9^*, #^
**3**	NI^#^	NI^#^
**4**	10.5 ± 4.3^*, #^	8.1 ± 2.1^*, #^
**5**	38.4 ± 5.5^*, #^	38.0 ± 5.8^*^
**6**	16.2 ± 4.7^*, #^	23.2 ± 4.3^*, #^
**7**	73.4 ± 5.1^*^	41.7 ± 4.4^*^
**2,4-D**^a^	100.0 ± 0.0^*, #^	100.0 ± 0.0^*, #^

Results were presented as the mean ± standard; a: 2,4-D = positive control; b:NI = not inhibited; the concentration for the test is 100 μg/mL; ^*^p < 0.05, significantly different from the control; ^#^*p* < 0.05, significantly different from the compound **7**

## Discussion

*Pinellia* Tenore is a well-known medicinal plant in China, and its tubers have high medicinal value. In this study, the diversity of endophytic fungi of *P. ternata* and *P. pedatisecta* was studied. A total of 77 fungi were isolated and identified by culture-dependent method and molecular biology sequencing for the first time. All fungi were distributed in 25 genera. Among them, 53 and 24 fungal strains were isolated from *P. ternata* and *P. pedatisecta*, respectively. The dominant genus from *P. ternata* and *P. pedatisecta* was *Fusarium*, which was also the dominant endophytic fungi from a medicinal plant *Ligusticum chuanxiong* Hort [31]. Howerver, endophytic fungal community composition of *Panax bipinnatifidus* var. *bipinnatifidus* was found to be significantly different from that of *P. ternata* and *P. pedatisecta*, with the dominant genus of the *Plectosphaerella*, *Paraphoma*, and *Fusarium* [32]. *Aspergillus* sp. [17–19, 33], *Chaetomium globosum* [34], and *Stachybotrys chartarum* [35] were reported to have been isolated from *P. ternata*. Among them, *C. globosum* and *S. chartarum* were different from the fungi isolated in this study. Therefore, many endophytic fungi of *P. ternata* might remain undiscovered.

Antibiotics are a class of compounds used to treat microbial infectious diseases, and they are widely used to treat human and animal diseases, as well as in agricultural production [36]. However, the overuse of antibiotics has led to a serious problem of antibiotic resistance, and the development of antibiotics is imminent [37]. Natural products are an important source of new antibiotics [38]. In this study, 77 strains of endophytic fungi from *P. ternata* and *P. pedatisecta* were evaluated for their antibacterial activity. The results showed that 21 strains had antibacterial effects. Furthermore, we investigated the secondary metabolites of PT09 and PP39 with good bioactive activities, which resulted in the isolation of alternariol monomethyl ether (**1**), alternariol (**2**), dehydroaltenusin (**3**), altertoxin II (**4**), terreic acid (**5**), terremutin (**6**), citrinin (**7**). Among them, metabolite **5** from *A. floccosus* PP39 showed strong inhibitory activity against all four tested bacteria in this study. The result was similar to it’s antibacterial activity against *E. coli*, *P. aeruginosa* and *Klebsiella pneumoniae* [39]. Further research into the antibacterial activity of metabolite **5** might be expected to develop novel antibiotics. Although metabolites **5** and **6** had similar structures, we found that metabolite **6** showed no obvious antibacterial activity. Compared with compound **5**, hydroxy at position 1 was oxidized to a carbonyl in compound **6**, suggesting that the 1-hydroxy was essential for the antibacterial activities [40]. Compound **7** exhibited strong antibacterial activity against *P. syringae* pv. *actinidiae* with the IZD of 26.0 mm and MIC value of 6.25 μg/mL. Furthermore, compound **7** has also been reported that it presented strong antibacterial activity against methicillin-resistant *S. aureus* and rifampicin-resistant *S. aureus* with the MIC values of 3.90 and 0.97 μg/mL, respectively [41]. However, there was evidence that compound **7** showed nephrotoxic, hepatotoxic, and carcinogenic activity [42, 43]. Therefore, it might not be suitable for developing antibiotic.

Herbicide resistance has become one of the most important issues in global crop production. New herbicides are needed to control weeds that are resistant to existing herbicides [44]. We investigated the herbicidal activity of fermentation broth of 77 endophytic fungi. The results revealed that 72 strains (93.5%) of endophytic fungi had herbicidal activity against *E. crusgalli*. It was reported that the secondary metabolites of 28 endophytic fungi from 21 plants also had great herbicidal effects [45]. Therefore, endophytic fungi might be a potential source of herbicidal resources [45].

The fermentation broth of both PT09 and PP39 had strong phytotoxic activity against *E. crusgalli* with the inhibition rate of more than 91%. However, the metabolites **1**–**7** from them had moderate or weak phytotoxic activities against *E. crusgalli*. Therefore, it was necessary to further explore the bioactive metabolites from strains PT09 and PP39 that inhibit *E. crusgalli* growth.

## Conclusions

In this study, 77 fungi were isolated and identified from roots, stems, leaves, and tubers of *P. ternata* and *P. pedatisecta.* Sequences analysis showed that all fungi were attached to the phyla Ascomycota and Basidiomycota, 68 strains of which were grouped into four classes. The most common endophytic fungi were *Fusarium* and *Aspergillus*. Antibacterial activities tests indicated that 21 endophytic fungi extracts exhibited antibacterial activities against at least one of the tested bacteria. Metabolite **5** from the *A. floccosus* PP39 exhibited outstanding antibacterial activities against *E. coli*, *M. tetragenus*, *S. aureus*, and *P. syringae* pv. *actinidiae* with the IZD of 36.0, 31.0, 33.7, 40.2 mm and MIC values of 1.56, 3.13, 1.56, 1.56 μg/mL respectively, which were better than or equal to positive gentamicin sulfate. The metabolite **7** also exhibited strong antibacterial activity against *P. syringae* pv. *actinidiae* with an IZD of 26.0 mm and MIC value of 6.25 μg/mL. Phytotoxic effects of 77 fungi on the radicle growth of *E. crusgalli* were investigated under laboratory conditions, and 22 fungi showed strong phytotoxic activity with the inhibition rate of 100%. In addition, the metabolite **7** had potent phytotoxic activity against *E. crusgalli* with the inhibition rate of 73.4% at the concentration of 100 μg/mL. In conclusion, this study showed that the endophytic fungi of *P. ternata* and *P. pedatisecta* held promise for the development of new antibiotic and herbicide resources.

## Methods

### Sample collection and microbial isolations

The healthy individuals of *P. ternata* and *P. pedatisecta* were collected from Hezhang County (27.13°N, 104.72°E, Bijie city, China) in March 2021. Five *P. pedatisecta* and *P. ternata* samples were selected and collected. These samples were immediately placed in autoclaved bags, labelled, and shipped to the laboratory in ice boxes. Then, they were stored at 4℃ and processed within 3 days.

All samples were washed thoroughly with sterile water, and the roots, tubers, stems, and leaves systems were separated from the individuals. The different plant tissues were cut into small pieces and soaked in 75% ethanol for 2 min, then in 5% sodium hypochlorite for 2–5 min, and followed by 75% ethanol for 1 min, respectively [46]. All samples were washed with sterile water for 2–3 times and then put on sterile filter paper to eliminate water. For control, the final sterile water rinse was spread on the plate and observed after incubation. The surface-sterilized explant fragments were homogenized separately in 1 mL of sterilized water. Then, the homogenates were serially diluted to 10^–1^ through 10^–4^ dilution, and 100 µL from each dilution was spread onto isolation media (Czapek–Dox Medium: NaNO_3_ 3 g, K_2_HPO_4_ 1 g, MgSO_4_ 0.5 g, KCI 0.5 g, FeSO_4_ 0.01 g, sugar 30 g, agar 15 ~ 20 g, distilled water 1000 mL, natural pH; Potato Dextrose Agar (PDA) Medium: potato 200 g, glucose 20 g, agar 15 ~ 20 g, distilled water 1000 mL, natural pH; MEA Medium: Malt extract 30 g, soybean peptone 3 g, agar 15 ~ 20 g, distilled water 1000 mL, natural pH). All isolation media were added with 50 μg/mL ampicillin and streptomycin. A single fungal colony from the isolation medium was inoculated into new PDA medium. The purified fungal strains were stored on PDA slope at 4℃.

### DNA Extraction and PCR Amplification

DNA sequencing was performed according to the previous methods with some modified [47]. Fungi were transferred into ME medium (20 g sucrose, 20 g malt extract, 1 g peptone, then added to 1 L with distilled water) and cultured at 28 ± 0.5℃ in Constant Temperature Incubator Shakers for 3 days. About 100 mg of fungal mycelium was used for gene DNA extraction. According to the manufacturer’s instructions, the DNeasy Plant Minikit (Qiagen, Germany) was utilized. The internal transcriptional spacer (ITS) of fungal ribosomal DNA was amplified using the forward primer ITS1 (5'-TCCGTAGGTGAACCTGCGG-3') and the reverse primer ITS4 (5'-TCCTCCGCTTATTGATATGC-3') [48]. The PCR products were sent to TSINGKE Biological Technology Corporation (Shandong, China) for purification and bi-directionally sequencing. Then, the obtained 5.8S rDNA sequences were uploaded to the National Center for Biotechnology Information (NCBI) database.

### Identification and phylogenetic analysis of the endophytic fungi

As previously reported, the affiliations of all resultant sequences returned from TSINGKE Biological Technology Corporation were identified by valid data in BLAST from NCBI database [47]. Sequence alignment and Neighbor-joining Phylogenetic Analysis were carried out using MEGA software version 7.0. Bootstrap analysis of tree construction built on 1000 replicates of sequence intensities to estimate neighbor-joining information [49].

### Diversity analyses of endophytic fungi from *P. ternata* and *P. pedatisecta*

The diversity of endophytic fungi was evaluated according to previously described methods [22]. The species richness was evaluated by the species richness index (*S*) and Margalef index (*D'*). The species diversity was assessed by the Shannon–Wiener index (*H'*), Simpson’s diversity index (*D*_*s*_), and Simpson’s dominant index (*λ*). The probability of interspecific encounters and species evenness was assessed by the probability of interspecific encounter (*PIE*) index and Pielou’s evenness index (*J*), respectively. All indexes were calculated by equation:

$${D}^{\mathrm{^{\prime}}}=(S-1)/\mathrm{ln}{N}_{t}$$

$${H}^{\mathrm{^{\prime}}}=-\sum_{i=1}^{s}{P}_{\mathrm{i}}\mathrm{ln}{P}_{\mathrm{i}},{ P}_{\mathrm{i}}={N}_{\mathrm{i}}/{N}_{\mathrm{t}}$$

$${D}_{\mathrm{s}}=1-\sum_{i=1}^{S}{P}_{\mathrm{i}}^{2}$$

$$\lambda =\sum_{i=1}^{S}{P}_{\mathrm{i}}^{2}$$

$$PIE=\sum_{i=1}^{s}({N}_{\mathrm{i}}/{N}_{\mathrm{t}})({N}_{\mathrm{t}}-{N}_{\mathrm{i}})/({N}_{\mathrm{t}}-1)$$

$$J=H\mathrm{^{\prime}}/{H}_{max} , {H}_{max}=\mathrm{ln}S$$

where *N*_*i*_ is the number of isolates belonging to the *i*th genus, *N*_*t*_ is the total number of endophytic fungal isolates’ in each tissue, *H* is *H'* of each tissue, and *S* is the number of total genera in each tissue.

### Preparation of extracts of fermentation broth of endophytic fungi

Each fungus was transferred to PDA medium and incubated at 28 ± 0.5℃ for 3–4 days. Then, fresh mycelia of each fungus were transferred to a 250 mL conical flask containing 150 mL of ME liquid medium and incubated in a constant temperature culture shaker rotating at 180 rpm for 7 days at 28 ± 0.5℃. The culture was passed through four layers of cotton gauze to obtain the fermentation broth, then the fermentation broth was extracted three times with ethyl acetate (EtOAc, 1:1, v/v). The crude fungal extracts were obtained by concentrating the ethyl acetate phase *in vacuo*.

### Isolation of compounds from PT09 and PP39

A total of 16 L of fermentation broth of PT09 was filtered and extracted three times with EtOAc (3 × 16 L) at room temperature [34]. The black-brown crude extract (2 g) was obtained by vacuum evaporation of the EtOAc phase. The crude fungal extract was separated by column chromatography (CC) using silica gel (SiO_2_: 200–300 mesh) and eluted with a stepwise gradient of CH_2_Cl_2_/MeOH (100:0–100:32, v/v) to provide seven primary fractions (Fr1 to Fr7). White needle-like crystals (compound **1**, 3 mg) were obtained from Fr1 by successive recrystallization with CH_2_Cl_2_/MeOH. Fr2 (CH_2_Cl_2_/MeOH, 100:1) was further separated on a silica gel chromatography column to obtain compounds **2** (2.5 mg) and **3** (2.5 mg). Compound **4** (1 mg) was isolated from Fr3 on a silica gel chromatography column with CH_2_Cl_2_/MeOH = 100:2.

The yellow–brown mixture of PP39 (5 g) was obtained using the above method. Compound **5** (24.5 mg) was isolated and purified from subfractions Fr1-5, which were obtained by silica gel column separation in component Fr1 (CH_2_Cl_2_/MeOH, 100:0). Compounds **6** (26.5 mg) and **7** (3.5 mg) were isolated and purified from component Fr3 (CH_2_Cl_2_/MeOH, 100:2) on a silica gel chromatography column.

### Structural elucidation of metabolites

The structures of all compounds were initially analyzed by ^1^H/^13^C Nuclear Magnetic Resonance (NMR) spectroscopy and High-Resolution Mass Spectrometry (HR-ESI–MS). ^1^H/^13^C NMR data were acquired using an Agilent DD2 600 Hz spectrometer (Agilent, USA), and chemical shifts were reported as parts per million (*δ*) by referring to tetramethylsilane (TMS) as an internal standard. HR-ESI–MS spectral data were collected on a TripeTOF 4600 mass analyzer (Bruker, USA).

### Antibacterial activity

The antibacterial activities of 77 fungal crude extracts and metabolites were assessed using the filter paper disk method [50]. Four tested bacteria (*E. coli*, *M. tetragenus*, *S. aureus*, and *P. syringae* pv. *actinidiae*) were used, and three of which (*E. coli*, *M. tetragenus*, and *S. aureus*) were cultured on trypticase soybean blood agar (TSBA) medium at 37℃. *P. syringae* pv. *actinidiae* was cultured on LB medium at 28℃. All tested crude extracts and metabolites were dissolved separately in acetone to obtain a concentration of 6 mg/mL. The gentamicin sulfate was used as positive control. All the tested crude extracts, metabolites and controls needed to be filtered by 0.22 μm sterile filter membrane. Next, sterile filter paper discs (6 mm in diameter) were added to 5 μL of the tested samples and then placed on the pre-prepared medium. Three replicates were established for each test. The petri dishes were incubated in a constant-temperature incubator for 24–36 h. The diameter of the inhibitory circle (in mm) was measured using the crossover method to assess the antibacterial activity.

The antibacterial activity of the minimum inhibitory concentration (MIC) of 96 well plates was determined by the continuous dilution method [20]. Compounds **1**–**7** were dissolved in dimethyl sulfoxide (DMSO) to prepare 10 mg/ml. The gentamicin sulfate was used as positive control.

### Phytotoxic assay

According to the methods described in previous literature [51], the phytotoxic activity of endophytic fungi was evaluated on radicle growth of *E. crusgalli*. The fungi were fermented in 150 mL of ME liquid medium at 28 ± 0.5℃ for 7 days to obtain the fermentation broth, which was filtered to remove mycelia. The *E. crusgalli* seeds were surface disinfected by soaking them in 5% sodium hypochlorite for 20 min. Then, the seeds were washed several times with deionized water. The seeds were cultured in an illuminating incubator at 28℃ until germination. Then, 30 pregerminated seeds were placed in 9 cm diameter Petri dishes on filter paper disks imbibed with 5.0 mL fungal fermentation broth. Radicle length was measured after 2–3 days, and distilled water was used as the negative control.

All compounds were evaluated for phytotoxic activity against *E. crusgalli* and *A. theophrasti* using Petri dish bioassay [52, 53]. *E. crusgalli* seeds were germinated using the method described above. The seeds of *A. theophrasti* were soaked in water at 60℃ for 30 min and transferred to a 40 mmol/L CaCl_2_ solution for 12 h. Then, the seeds were surface disinfected according to the above method and transferred to a 28℃ illuminating incubator until germination. The compounds were dissolved in acetone and diluted to 100 μg/mL with 0.1% aqueous Tween-80. The bioassay of the phytotoxic activity for compounds was the same as that of fermentation broth. 2,4-Dichlorophenoxyacetic acid (2,4-D) was used as the positive control.

### Statistical analysis

Statistical differences were analyzed by one-way ANOVA with post-hoc LSD test and *t*-test. Values were considered significantly different when P-value was less than 0.05.

## Supplementary Information

**Additional file 1.**

## Data Availability

The datasets generated and/or analysed during the current study are available in the NCBI repository, ON677855-ON677931.
