# Supplementary material for: Diversity, antibacterial and phytotoxic activities of culturable endophytic fungi from Pinellia pedatisecta and Pinellia ternata
Source: BMC Microbiol. 2023 Jan 28;23:30. doi: 10.1186/s12866-022-02741-5 (PMC9883868; doi:10.1186/s12866-022-02741-5)
Supplement: Supplementary file 1 — Additional file 1. [file 12866_2022_2741_MOESM1_ESM.docx]

Supplementary Material

Diversity, antibacterial and phytotoxic activities of culturable endophytic fungi from *Pinellia pedatisecta* and *Pinellia ternata*

Kun Kong^1^, Zhongdi Huang^1^, Shuping Shi^1^, Weidong Pan^2*^, Yinglao Zhang^1*^

^1^School of Life Sciences, Anhui Agricultural University, Hefei 230036, China,

^2^State Key Laboratory of Functions and Applications of Medicinal Plants, Guizhou Medical University, Guiyang 550014, China.

* **Correspondence:**Yinglao Zhang (zhangyl@ahau.edu.cn); Weidong Pan (wdpan@163.com)

**Table S1.** Antibacterial activities of 77 fungal crude extracts from *P. ternata* and *P. pedatisecta* (mm).

| Strains | S. aureus | M. tetragenus | E. coli | Psa |
| --- | --- | --- | --- | --- |
| PT01 | NI | NI | NI | NI |
| PT02 | 9.0 ± 0.4^*, #^ | 9.5 ± 1.1^*, #^ | 12.2 ± 0.6^*, #^ | 7.7 ± 0.5^*, #^ |
| PT03 | NI | NI | NI | NI |
| PT04 | NI | NI | NI | NI |
| PT07 | NI | NI | NI | NI |
| PT08 | NI | NI | NI | NI |
| PT09 | 9.5 ± 0.0^*, #^ | NI | NI | 10.0 ± 0.0^*, #^ |
| PT10 | NI | NI | NI | NI |
| PT11 | NI | NI | NI | NI |
| PT12 | NI | NI | NI | NI |
| PT13 | NI | NI | NI | 9.3 ± 0.5^*, #^ |
| PT14 | NI | NI | NI | NI |
| PT15 | NI | NI | NI | NI |
| PT16 | NI | NI | NI | NI |
| PT17 | NI | NI | NI | NI |
| PT25 | NI | NI | NI | NI |
| PT26 | NI | NI | NI | NI |
| PT27 | NI | NI | NI | NI |
| PT28 | NI | NI | NI | NI |
| PT29 | NI | NI | NI | NI |
| PT30 | NI | NI | NI | NI |
| PT31 | NI | NI | NI | NI |
| PT31-1 | NI | NI | NI | NI |
| PT31-3 | NI | NI | NI | NI |
| PT31-4 | NI | NI | NI | NI |
| PT31-5 | NI | NI | NI | NI |
| PT32 | 16.3 ± 0.4^*, #^ | NI | NI | 14.7 ± 0.5^*, #^ |
| PT34 | 10.0 ± 0.8^*, #^ | 8.7 ± 0.9^*, #^ | 7.0 ± 0.4^*, #^ | NI |
| PT54 | NI | NI | NI | NI |
| PT55 | 7.0 ± 0.0^*, #^ | NI | NI | NI |
| PT56 | NI | NI | NI | 15.2 ± 0.2^*, #^ |
| PT58 | 10.0 ± 0.8^*, #^ | 7.5 ± 0.4^*, #^ | 9.0 ± 0.8^*, #^ | 13.3 ± 0.5^*, #^ |
| PT59 | NI | NI | NI | NI |
| PT60 | 7.3 ± 0.2^*, #^ | NI | 7.3 ± 0.5^*, #^ | NI |
| PT61 | NI | NI | NI | NI |
| PT62 | NI | NI | NI | NI |
| PT63 | NI | NI | NI | NI |
| PT64 | NI | NI | NI | NI |
| PT65 | NI | NI | NI | NI |
| PT66 | NI | NI | NI | NI |
| PT67 | NI | NI | NI | NI |
| PT68 | NI | NI | NI | NI |
| PT70 | NI | NI | NI | NI |
| PT71 | NI | NI | NI | NI |
| PT72 | NI | NI | NI | NI |
| PT74 | NI | NI | NI | NI |
| PT77 | NI | NI | NI | NI |
| PT78 | NI | NI | NI | NI |
| PT80 | NI | NI | NI | NI |
| PT81 | NI | NI | NI | NI |
| PT82 | NI | NI | NI | 15.7 ± 0.5^*, #^ |
| PT83 | NI | NI | NI | 20.0 ± 0.8^*, #^ |
| PT84 | NI | NI | NI | NI |
| PP33 | NI | NI | NI | NI |
| PP35 | 9.3 ± 1.3^*, #^ | 8.5 ± 0.5^*, #^ | 11.3 ± 0.9^*, #^ | 9.7 ± 0.5^*, #^ |
| PP36 | NI | NI | NI | NI |
| PP37 | 8.0 ± 0.8^*, #^ | 8.0 ± 0.5^*, #^ | 10.0 ± 0.8^*, #^ | 12.0 ± 0.8^*, #^ |
| PP38 | 9.3 ± 1.2^*, #^ | 10.3 ± 0.9^*, #^ | NI | 11.3 ± 0.9^*, #^ |
| PP39 | 20.0 ± 0.8^*^ | 14.2 ± 0.2^*^ | 15.2 ± 0.8^*^ | 14.0 ± 0.8^*^ |
| PP40 | NI | NI | NI | NI |
| PP41 | NI | NI | NI | 9.0 ± 0.8^*, #^ |
| PP42 | NI | NI | NI | NI |
| PP43 | NI | NI | NI | NI |
| PP44 | NI | NI | NI | 8.3 ± 0.5^*, #^ |
| PP45 | NI | NI | NI | NI |
| PP46 | NI | NI | NI | NI |
| PP47 | NI | NI | NI | NI |
| PP48 | NI | NI | NI | NI |
| PP49 | NI | NI | NI | NI |
| PP50 | NI | NI | NI | NI |
| PP51 | 14.2 ± 1.2^*, #^ | NI | NI | 9.5 ± 0.4^*, #^ |
| PP52 | NI | NI | NI | NI |
| PP53 | NI | NI | NI | NI |
| PP69 | NI | NI | NI | 8.3 ± 0.5^*, #^ |
| PP73 | NI | 7.5 ± 0.5^*, #^ | NI | 12.0 ± 0.0^*, #^ |
| PP75 | NI | NI | NI | NI |
| PP76 | 7.0 ± 0.0^*, #^ | 7.5 ± 0.5^*, #^ | NI | 7.7 ± 0.5^*, #^ |
| Gentamicin sulfate^a^ | 21.7 ± 0.6^#^ | 25.7 ± 0.9^#^ | 26.7 ± 0.5^#^ | 24.3 ± 0.5^#^ |

^a^Gentamicin sulfate = positive control; results are presented as the mean ± standard; “NI” means not inhibited; the concentration for the test is 30 µg/filter paper; ^*^*p* < 0.05, significantly different from the control; ^#^*p* < 0.05, significantly different from the strain PP39.

**Figure S1.** The HR–ESI-MS of compound **1**.

**
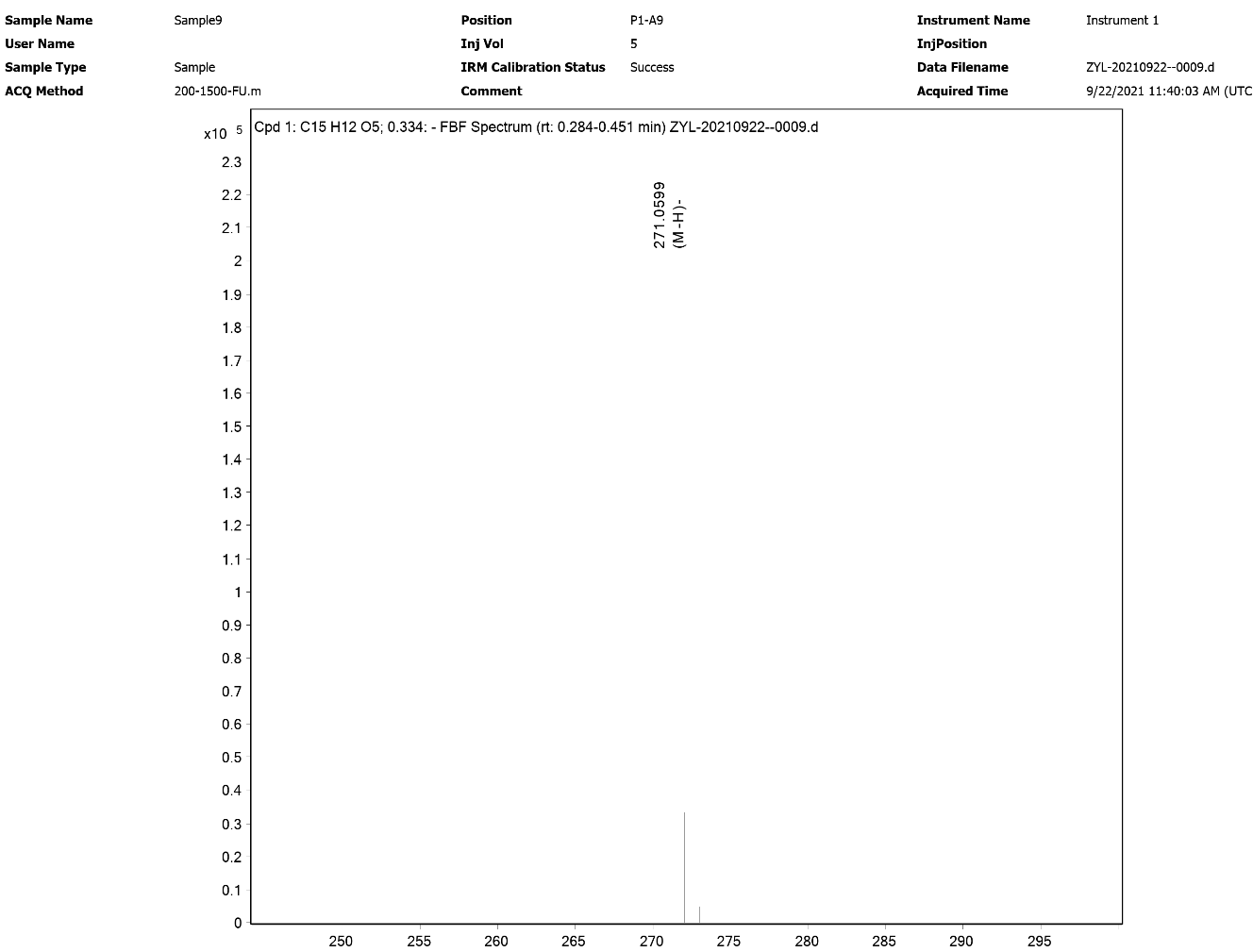
**

**Figure S2.** The ^1^H NMR of compound **1**.


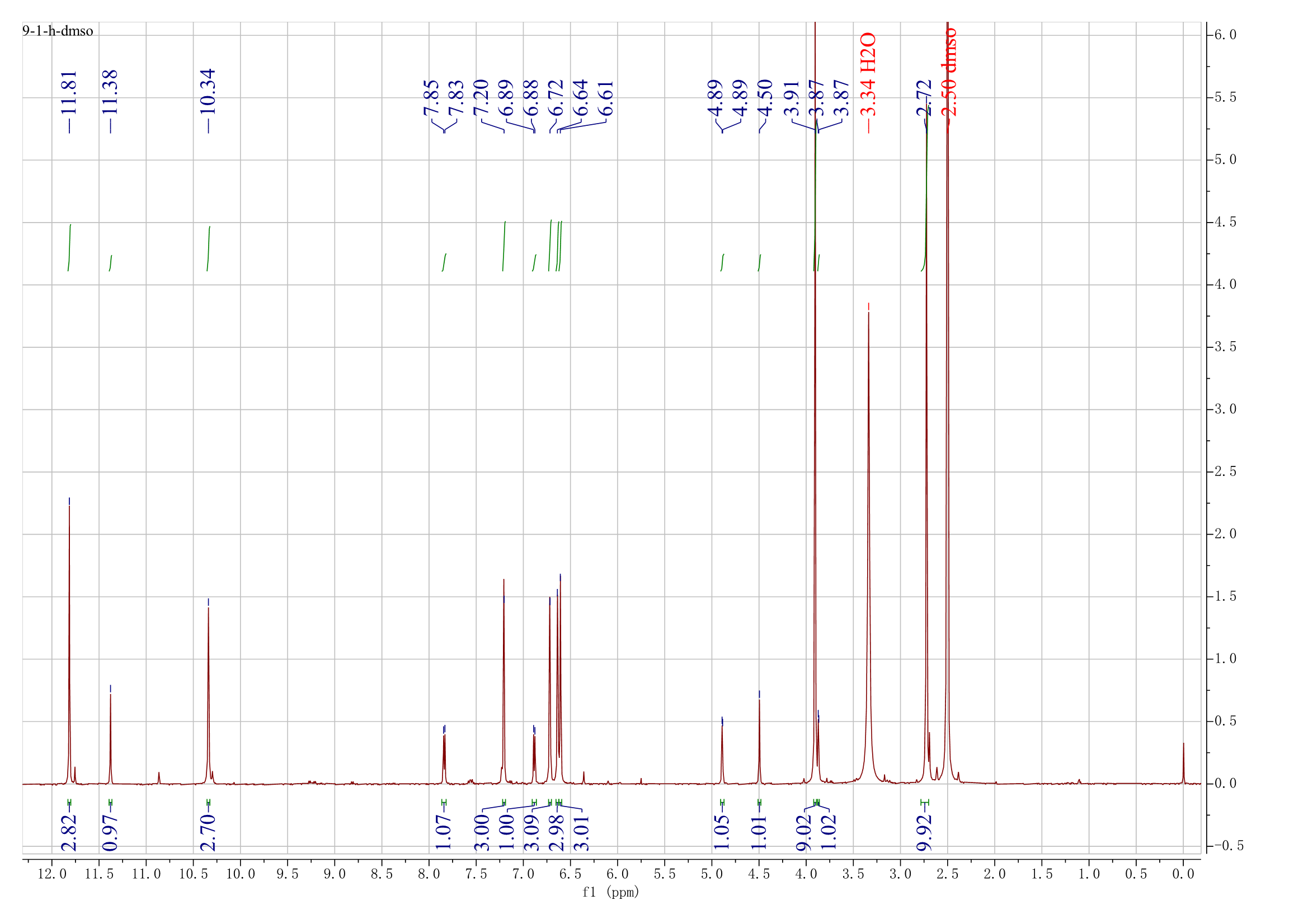


**Figure S3.** The HR–ESI-MS of compound **2**.


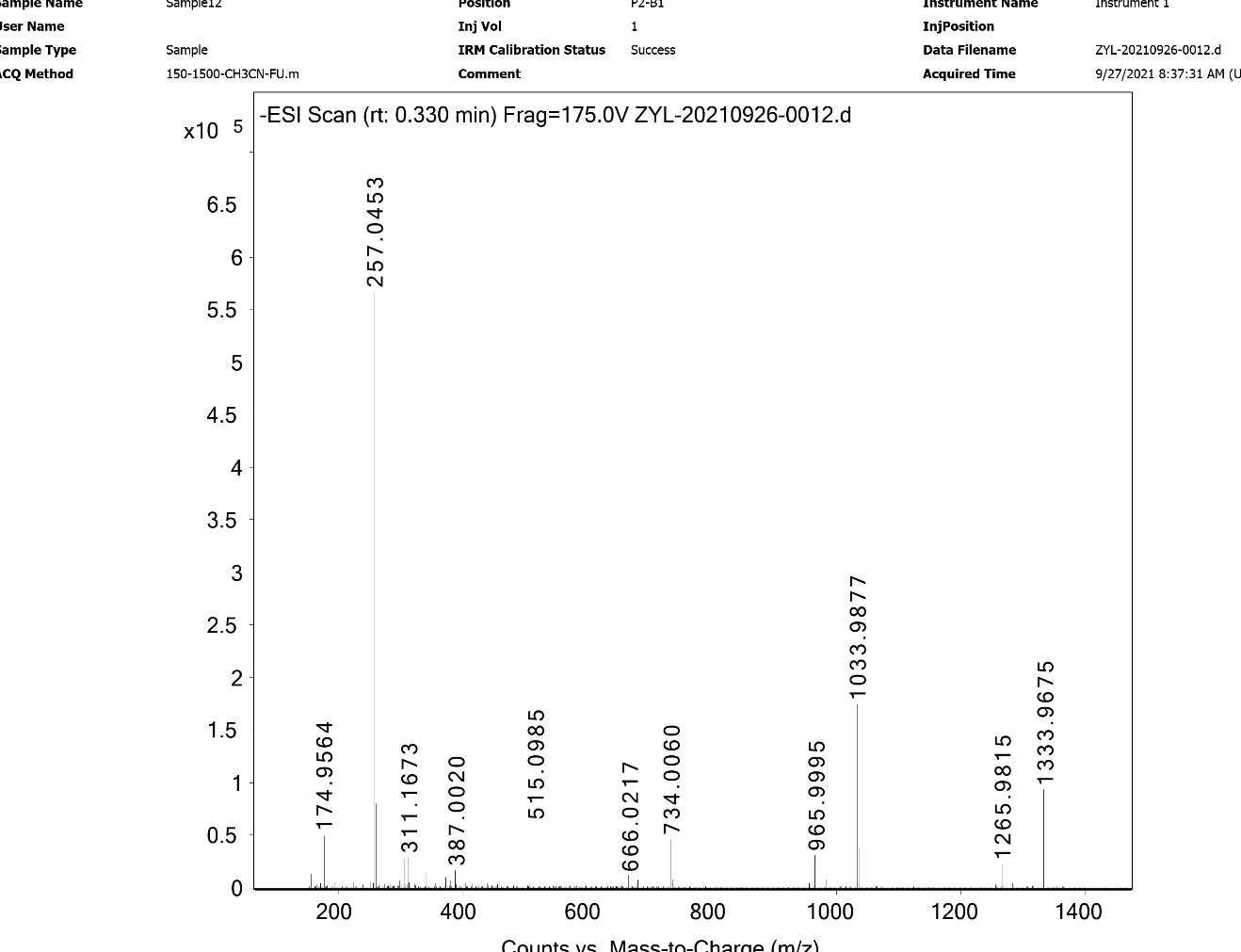


**Figure S4.** The ^1^H NMR of compound **2**.


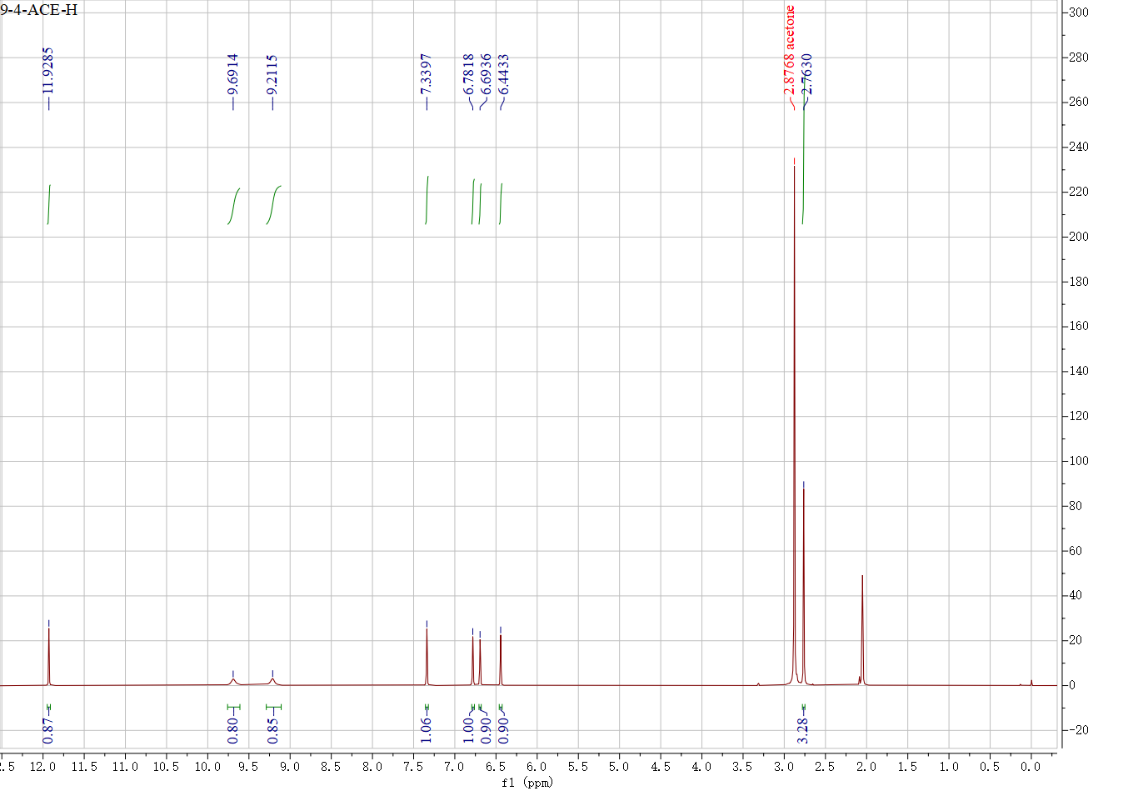


**Figure S5.** The HR-ESI-MS of compound **3**.


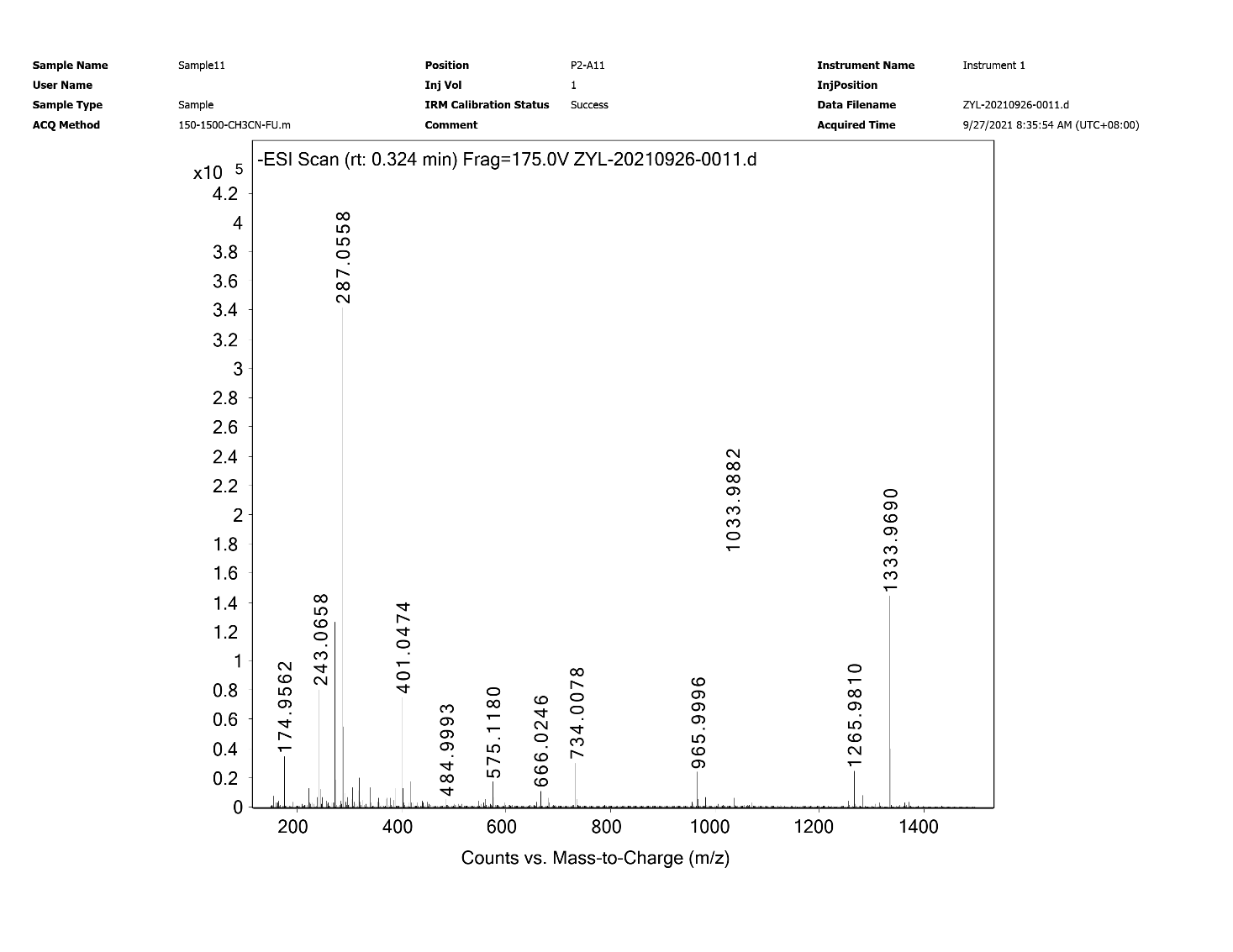


**Figure S6.** The ^1^H NMR of compound **3**.


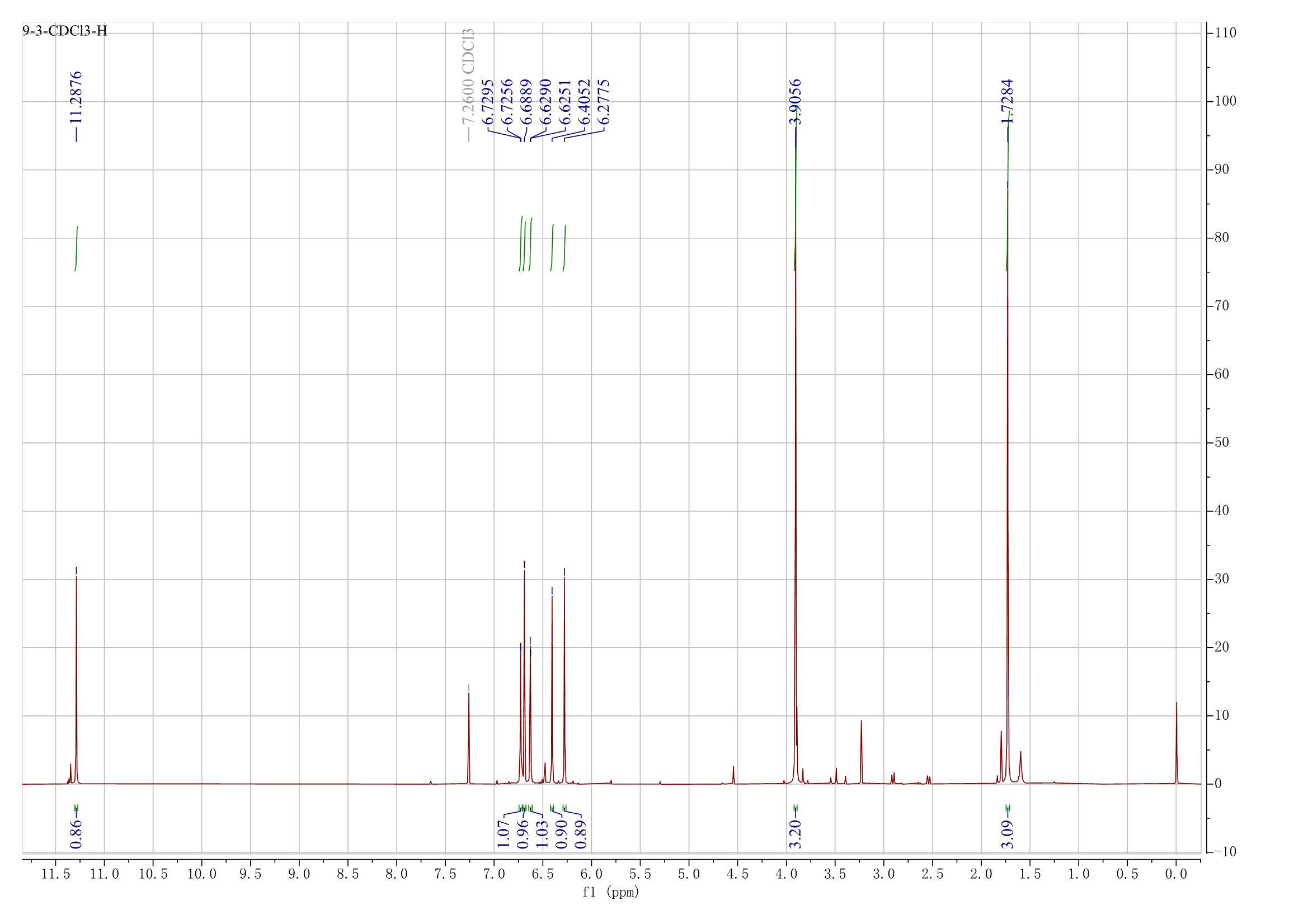


**Figure S7.** The ^13^C NMR of compound **3**.


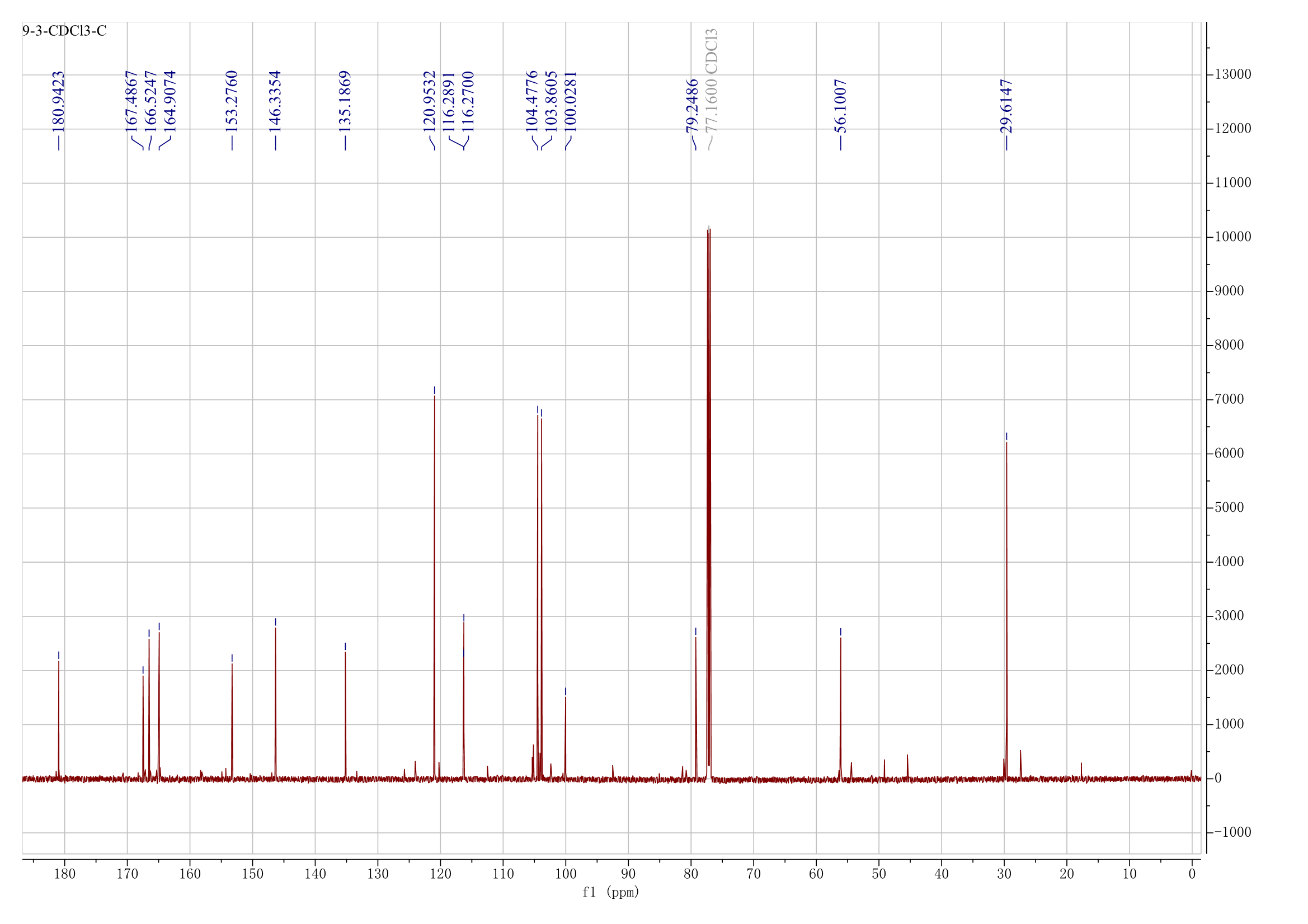


**Figure S8.** The HR-ESI-MS of compound **4**.


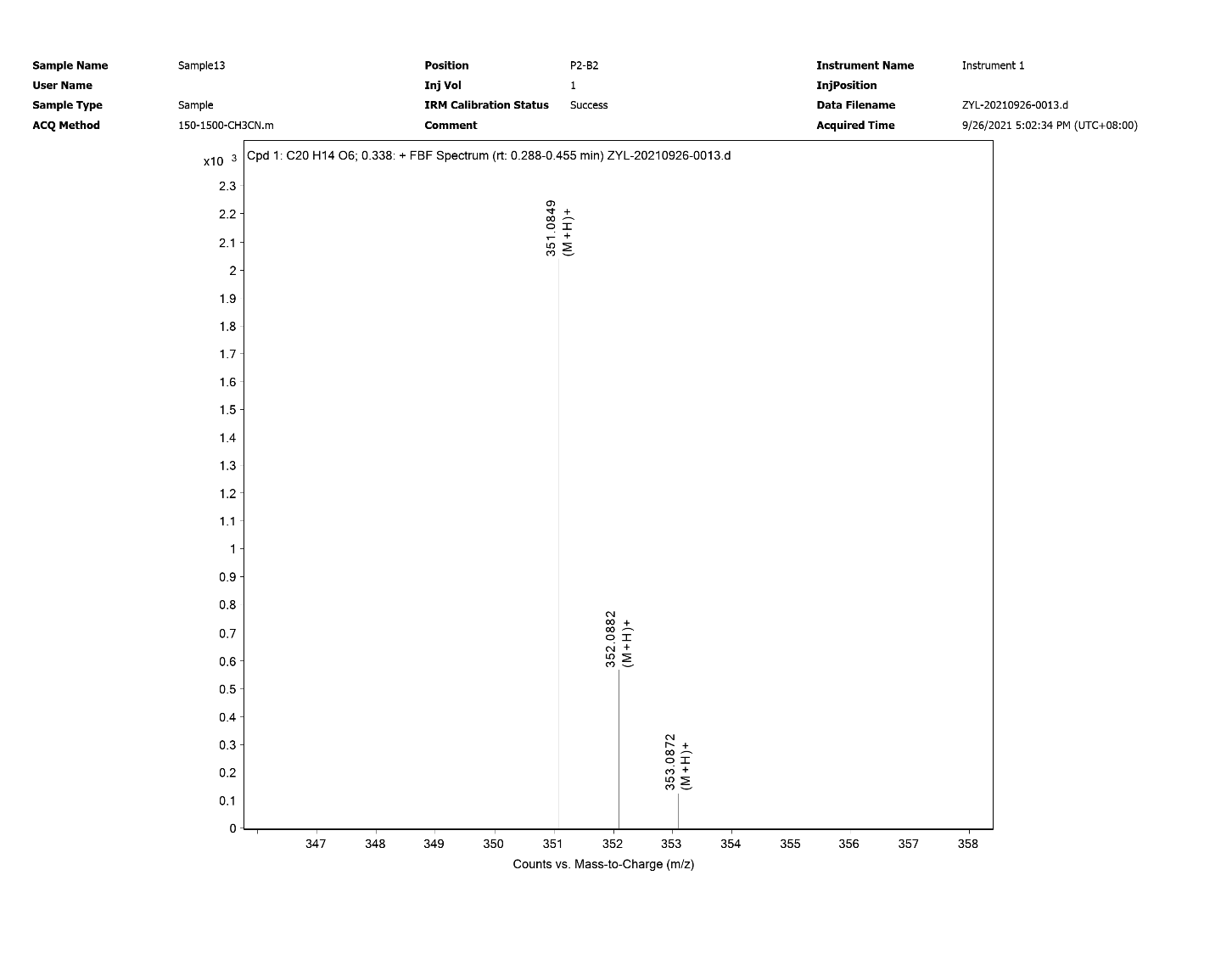


**Figure S9.** The ^1^H NMR of compound **4**.


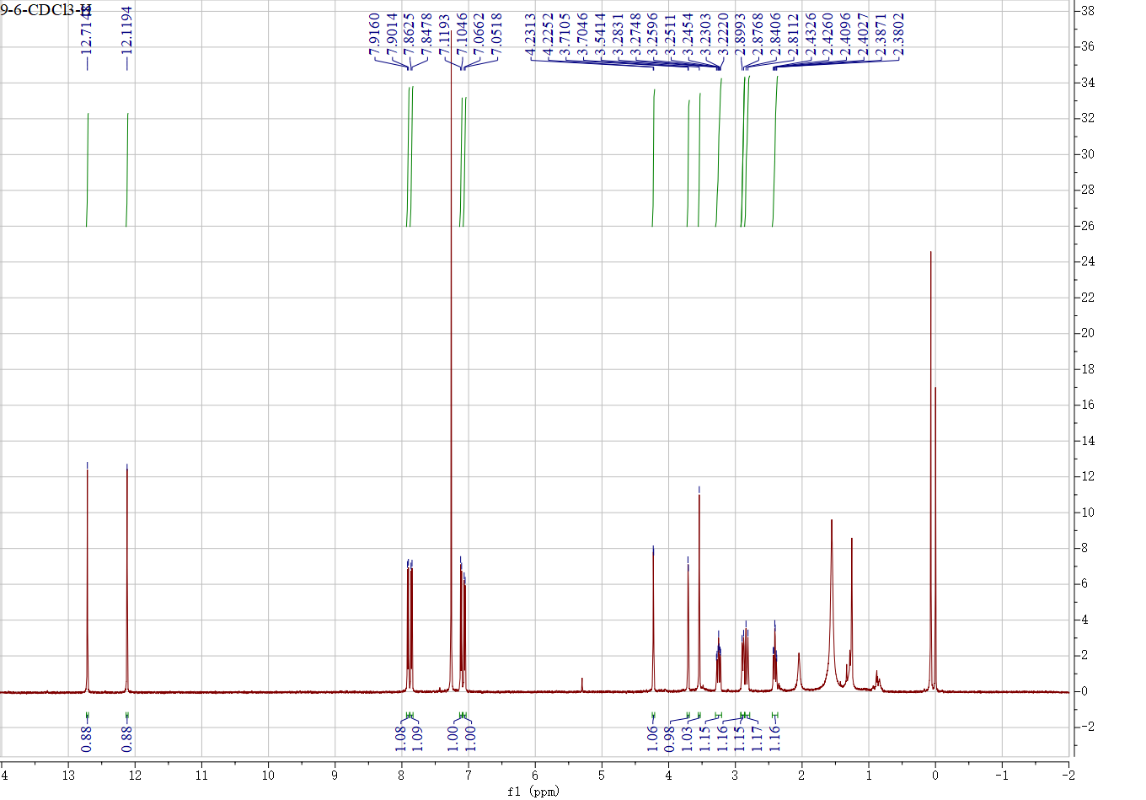


**Figure S10.** The ^13^C NMR of compound **4**.


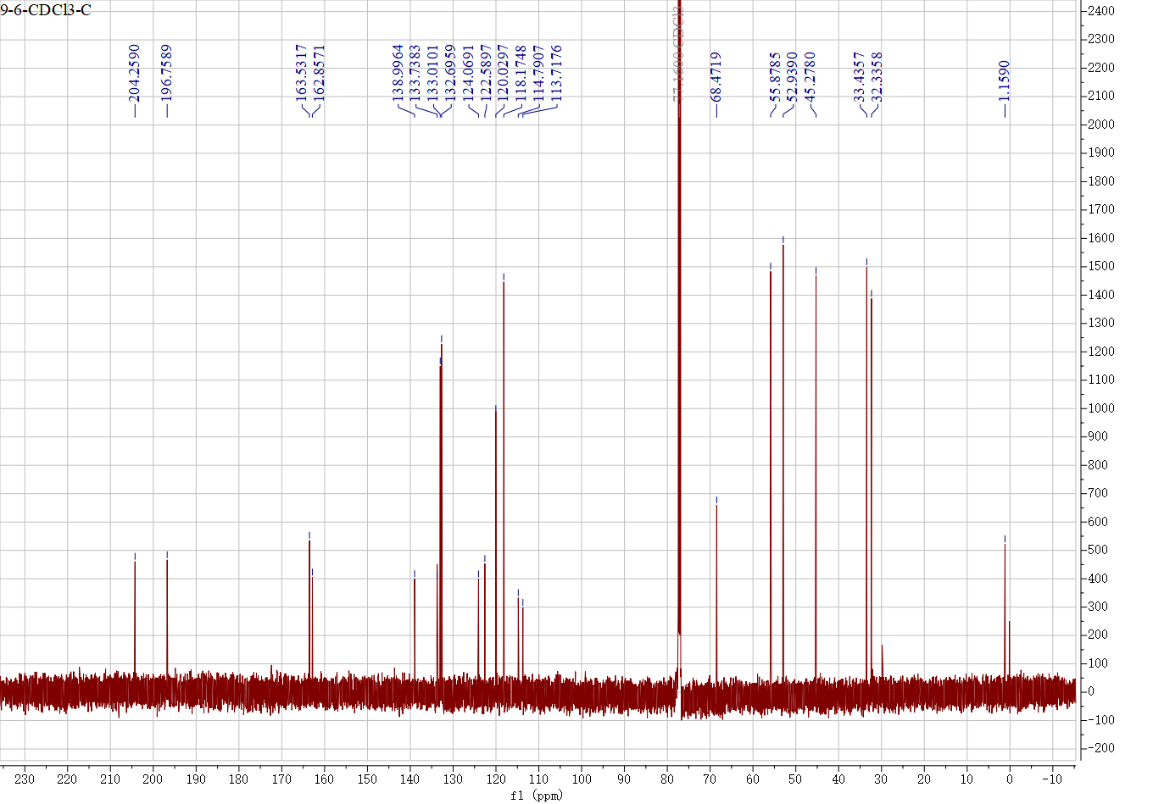


**Figure S11.** The HR-ESI-MS of compound **5**.


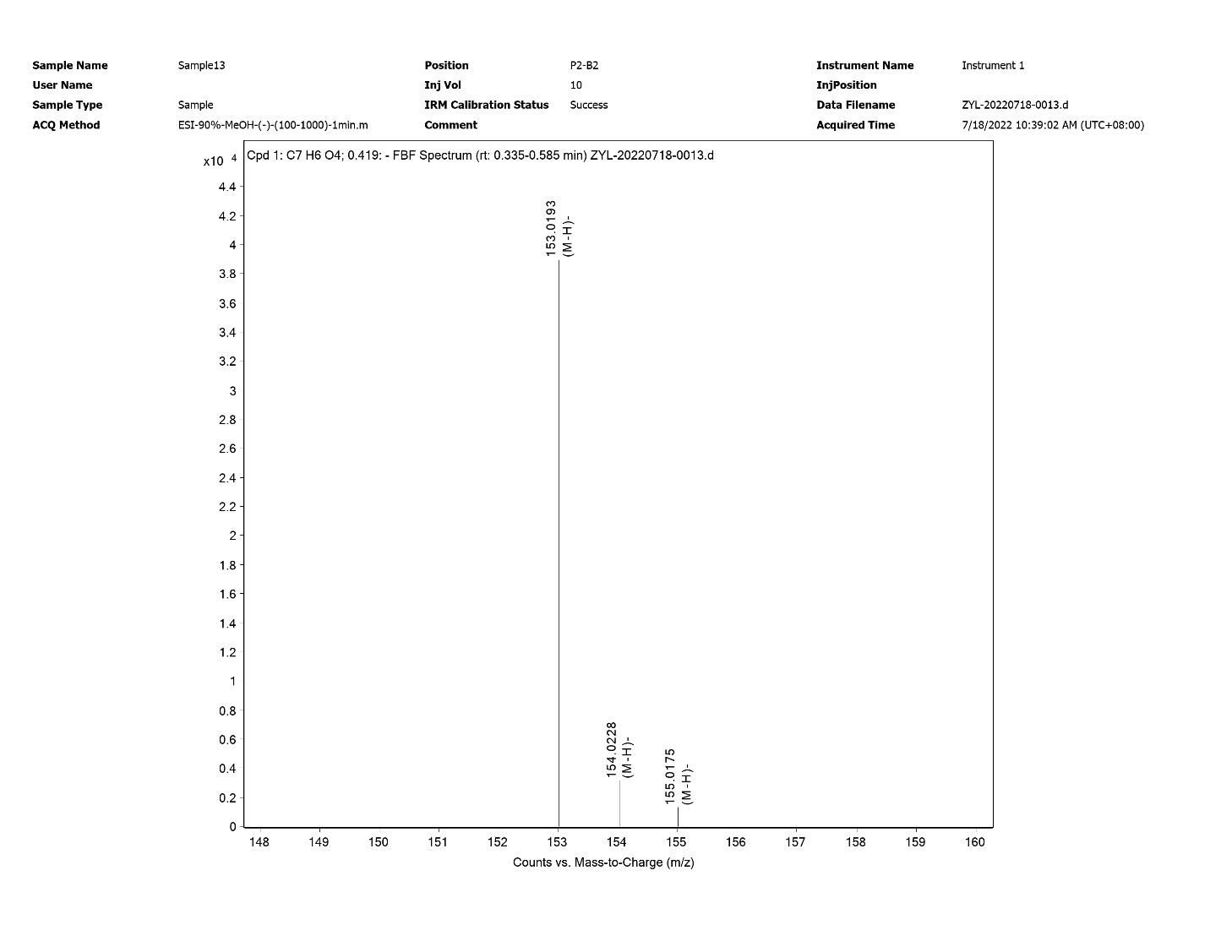


**Figure S12.** The ^1^H NMR of compound **5**.


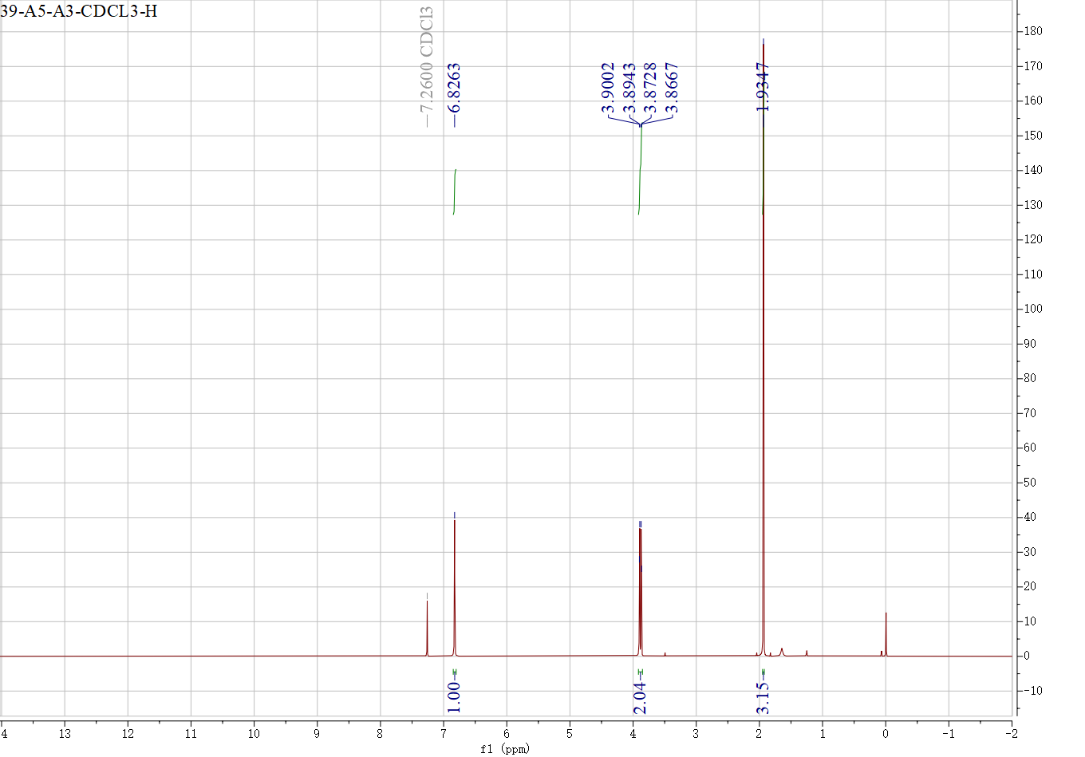


**Figure S13.** The ^13^C NMR of compound **5**.


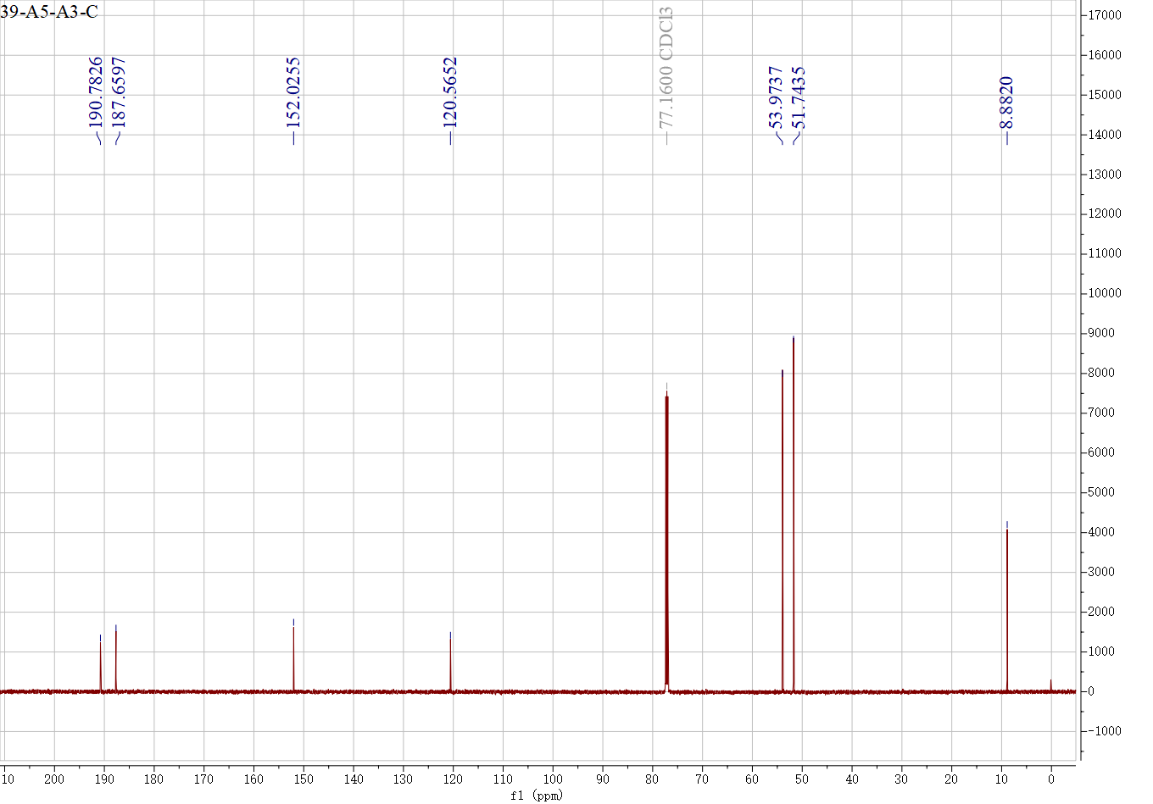


**Figure S14.** The HR-ESI-MS of compound **6**.


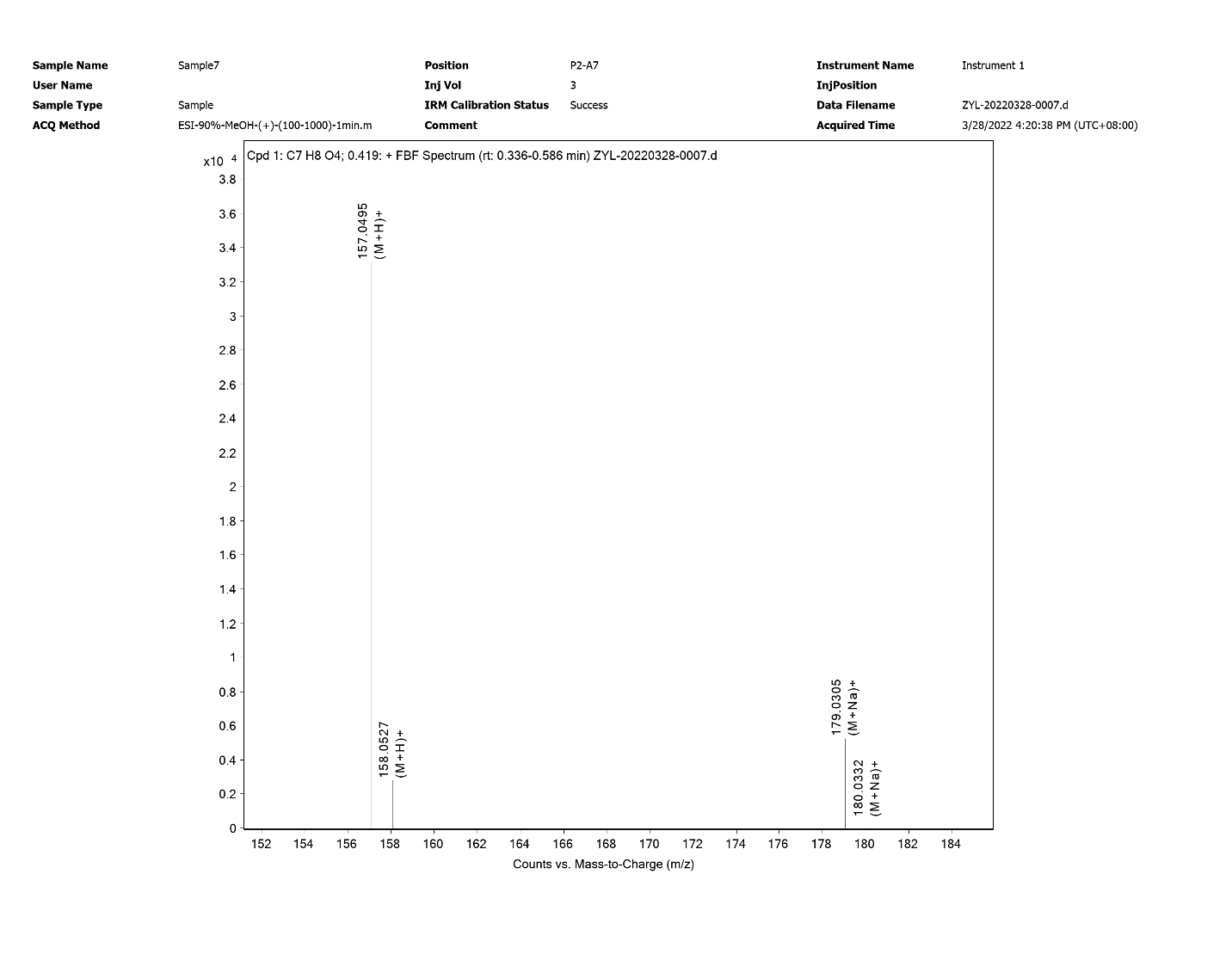


**Figure S15.** The ^1^H NMR of compound **6**.


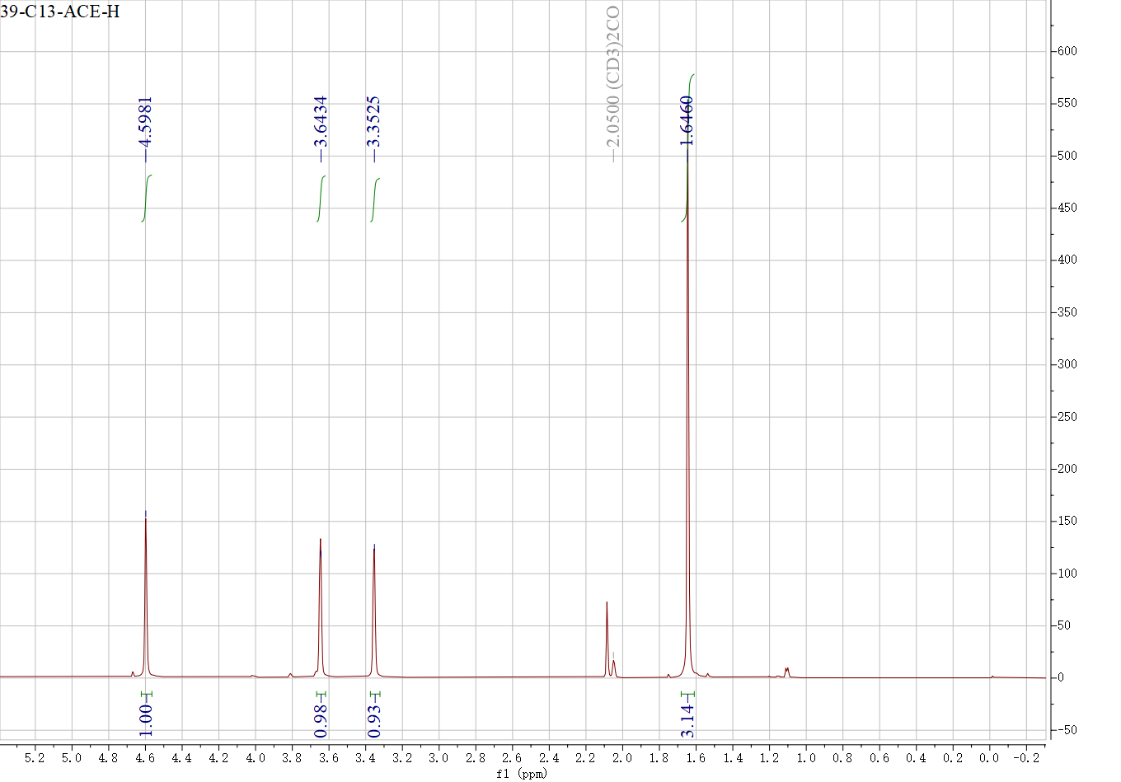


**Figure S16.** The ^13^C NMR of compound **6**.


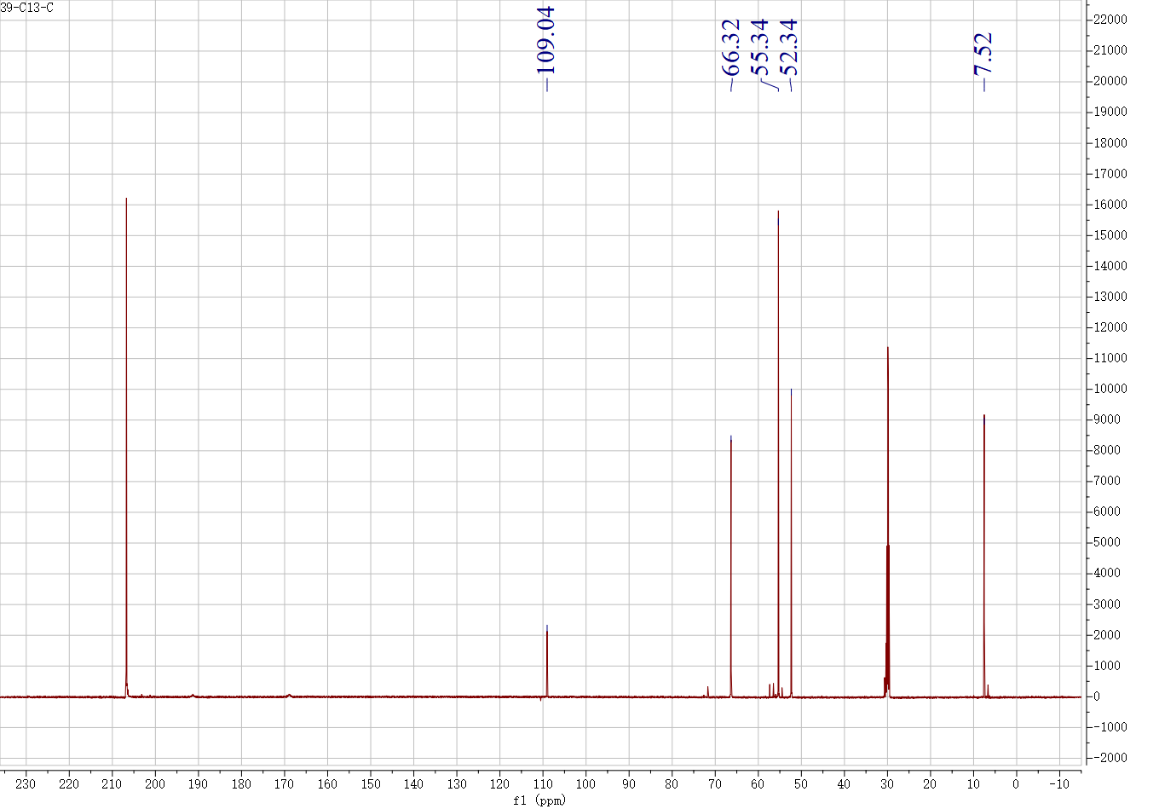


**Figure S17.** The HR-ESI-MS of compound **7**.


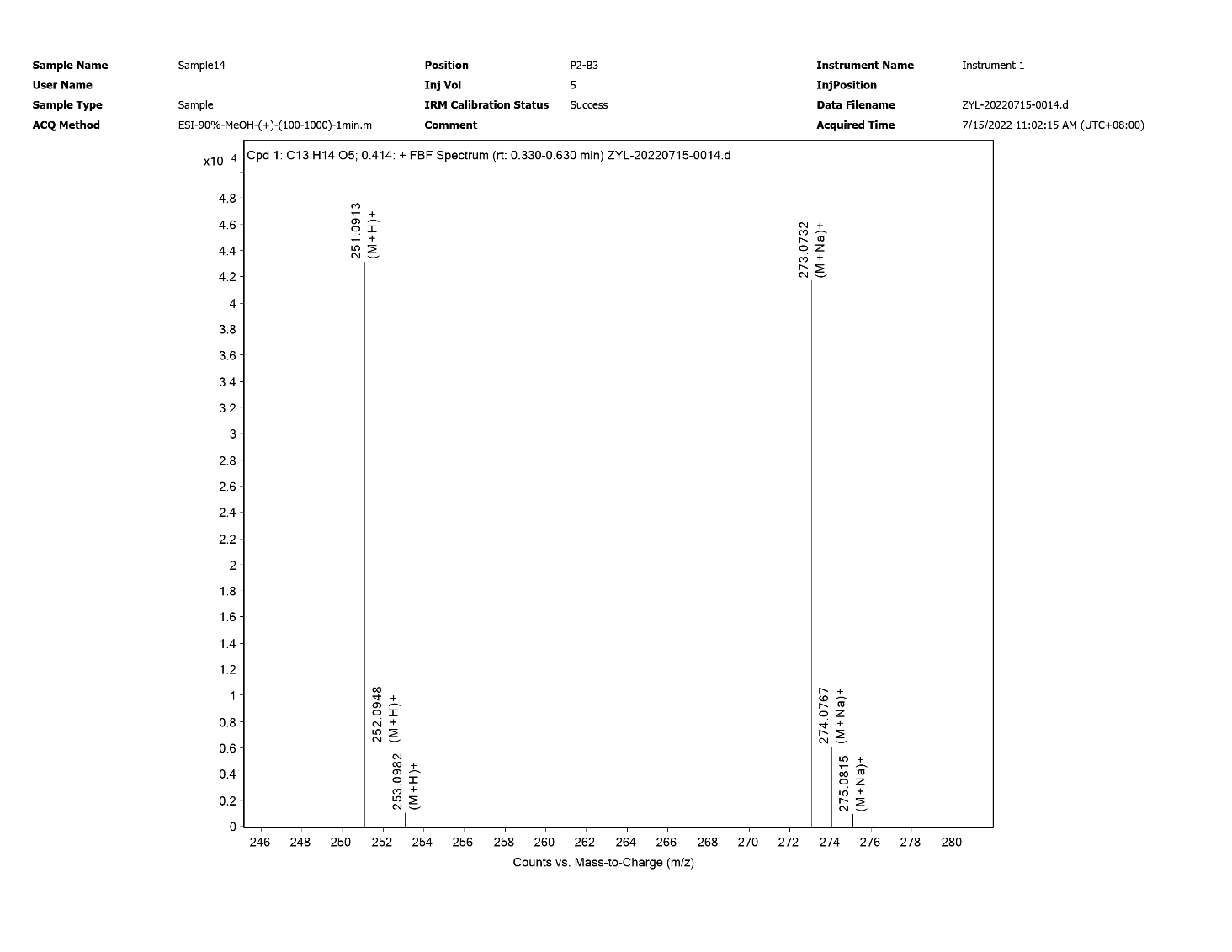


**Figure S18.** The ^1^H NMR of compound **7**.

**
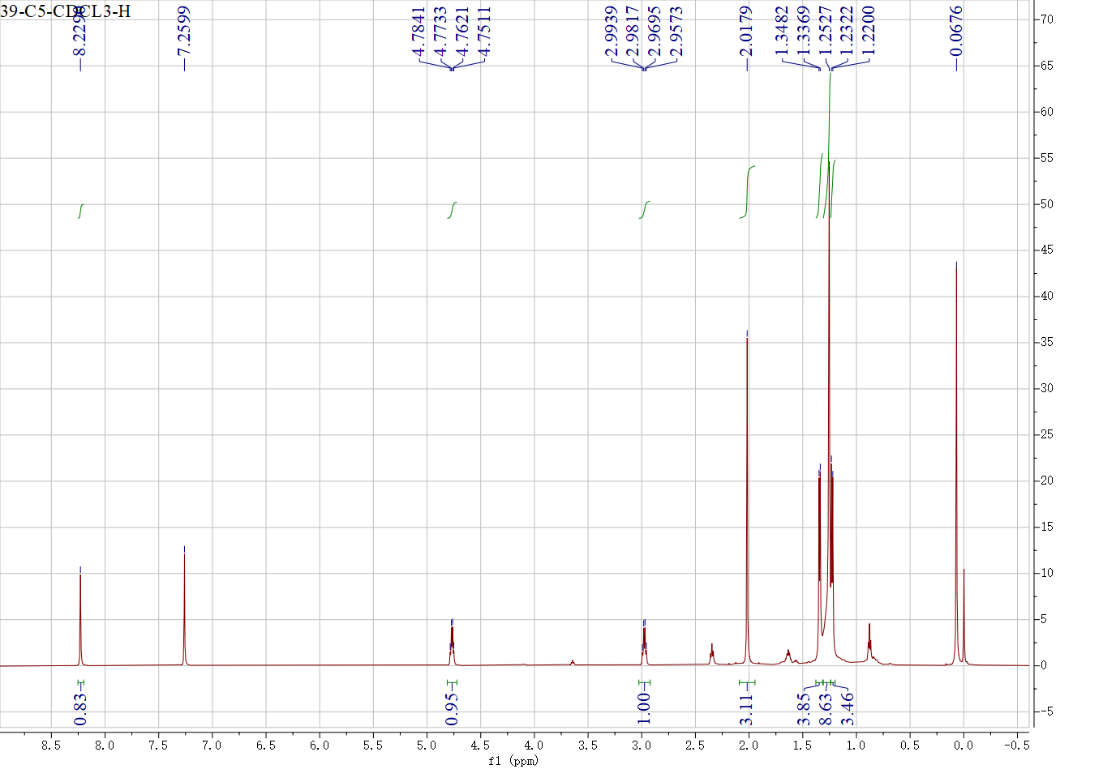
**

**Figure S19.** The ^13^C NMR of compound **7**.

**
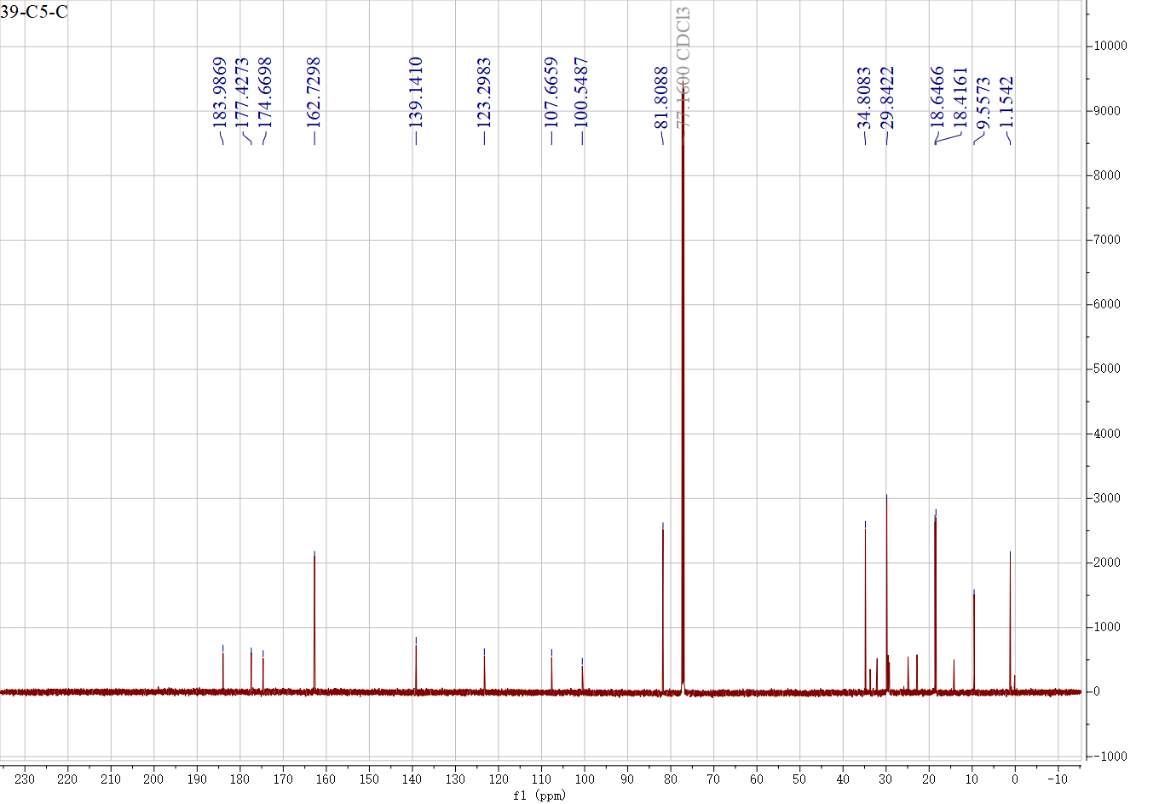
**
